# Quorum sensing gene *abaI* enhancing capsule production and pathogenicity of *Acinetobacter baumannii*

**DOI:** 10.3389/fmicb.2026.1865788

**Published:** 2026-09-01

**Authors:** Zhilang Qiu, Yu Zheng, Kexin Yuan, Peiling Zhao, Guanfeng Wu, Yang Zeng, Huijun Cao, Xuejun Zhao, Liang Hong, Guo Guo, Fei Mo, Yuping Wang, Jian Peng

**Affiliations:** 1 Center for Clinical Laboratories, The Affiliated Hospital of Guizhou Medical University, Guiyang, China; 2 Guizhou Key Laboratory of Microbio and Infectious Disease Prevention & Control, School of Biology and Engineering (School of Modern Industry for Health and Medicine)/School of Basic Medical Sciences, Guizhou Medical University, Guiyang, China; 3 Engineering Research Center for Bio-Perception Materials of Guizhou Province, Guiyang, China; 4 Engineering Research Center of Health Medicine Biotechnology of Institution of Higher Education of Guizhou Province, Guiyang, China; 5 Department of Clinical Laboratory, Guizhou Provincial People's Hospital, Guiyang, China

**Keywords:** *abaI*, *Acinetobacter baumannii*, inflammatory response, quorum sensing, virulence

## Abstract

**Introduction:**

*Acinetobacter baumannii* (*A. baumannii*) is a Gram-negative coccobacillus that can cause a variety of infectious diseases. The Quorum Sensing (QS) system is a widespread mechanism regulating group behavior in bacteria, playing a crucial role in the virulence regulation of several Gram-negative pathogenic bacteria. The aim of this study was to investigate the effects of the *A. baumannii* quorum sensing system on capsule production and pathogenicity.

**Methods:**

In this work, a clinical carbapenem-resistant *A. baumannii* with mucoid colonies (CRAB110) was used to construct an *abaI* knockout strain (CRAB110Δ*abaI*) using CRISPR/Cas9 system. Afterwards, changes in capsule production, bacterial virulence, and host immune responses were evaluated by assessing bacterial phenotypes, physiology, gene expression, and effects on the host.

**Results:**

The results showed that CRAB110Δ*abaI* significantly affected capsule production, virulence, and immunity compared with CRAB110. Mice infected with CRAB110Δ*abaI* exhibited a reduction in inflammation compared to those infected with CRAB110. A comparison of the transcriptomes of CRAB110 and CRAB110Δ*abaI* identified differentially expressed genes related to polysaccharide synthesis (*wza*, *wzb*, *wzc*), galactose metabolism (*GalM*), ABC transport, and amino acid metabolism. Mouse lung transcriptomic and cellular Western blot data indicated that quorum sensing (QS) exerts its pro-inflammatory effect through *Acinetobacter baumannii* by influencing the IL-17 and NF-κB pathways.

**Conclusion:**

This study suggests that the *abaI* gene boosts capsule production and pathogenicity of *A. baumannii*, providing new insights for clinical management of drug-resistant *A. baumannii* diseases and a foundation for new antimicrobial drugs.

## 1 Introduction

*Acinetobacter baumannii* (*A. baumannii*) is a nonfermenting, strictly aerobic, Gram-negative, bacillus ubiquitously distributed in natural environments (Iovleva et al., 2025). Epidemiological investigations have elucidated its capacity to induce a spectrum of infections, including ventilator-associated pneumonia (VAP), bloodstream infections, and urinary tract infections (AbdelHalim et al., 2024). Among these, VAP caused by *A. baumannii* represents the most prevalent nosocomial infection, predominantly afflicting intensive care unit (ICU) patients requiring mechanical ventilation (Kaye et al., 2023). Clinical data reveal that the mortality rate of such VAP ranges from 40 to 70% (Ibrahim et al., 2021; Ayoub Moubareck and Hammoudi Halat, 2020). Carbapenem antibiotics, renowned for their broad-spectrum activity and potent bactericidal efficacy, have long been the first-line therapy for multidrug-resistant *A. baumannii* infections. However, their rampant utilization has precipitated the emergence of severe drug resistance in *A. baumannii* (Zhang et al., 2021). Faced with substantial mortality attributable to carbapenem-resistant *A. baumannii*, it is critically important to elucidate the mechanisms underlying host infection by carbapenem-resistant *A. baumannii*.

*A. baumannii* was conventionally regarded as a low-virulence pathogen, but accumulating research has identified multiple virulence factors contributing to its pathogenicity, thereby revolutionizing the prevailing perception (Wu et al., 2025). These virulence determinants encompass penicillin-binding proteins (PBPs), outer membrane protein OmpA, lipopolysaccharides (LPS), capsular polysaccharides (CPS), phospholipases, outer membrane vesicles (OMVs), and protein secretion systems (Lucidi et al., 2024; Gautam et al., 2023). These factors may confer adaptive advantages at various stages of pathogenesis, spanning from evasion of host immune surveillance to host cell adhesion, internalization, and induction of apoptosis.

The Quorum Sensing (QS) system, a ubiquitous regulatory mechanism in bacteria, modulates collective behaviors by perceiving population density and plays a pivotal role in regulating bacterial virulence (Mayer et al., 2023). Its centrality in virulence regulation has been substantiated in numerous prominent pathogenic and opportunistic pathogenic bacteria, including *Pseudomonas aeruginosa* and *Escherichia coli* (Mayer et al., 2023; Venkateswaran et al., 2023). The QS system of *A. baumannii* primarily comprises *abaI* (signal synthase), AbaR (transcriptional regulator), and AHL (acyl-homoserine lactone) (Jiang et al., 2024). The signal molecule synthase gene *abaI* is instrumental in AHL biosynthesis, utilizing intracellular S-adenosylmethionine (SAM) and acyl-acyl carrier protein as substrates to synthesize acylated homoserine lactone (AHL) signal molecules, which are subsequently secreted extracellularly (Jiang et al., 2024; Sun et al., 2021). Upon reaching a threshold concentration, they interact with the transcriptional regulator AbaR, thereby modulating corresponding gene expression alterations within the bacterial population. Existing studies suggest that QS regulates motility (Rajapaksha et al., 2023), antibiotic resistance (Cui et al., 2025), and biofilm formation (Bell and Muniyan, 2025), with these phenotypic and functional modifications playing pivotal roles in *A. baumannii* pathogenicity. Additionally, QS influences eukaryotic host cells and modulates immune responses. For instance, QS molecules produced by *Pseudomonas aeruginosa* can modulate the host TNFR/caspase 8 pathway through direct activation of host TNFR1 signaling, thereby dampening the host inflammatory response (Song et al., 2019). This further underscores the significance of the QS system in regulating host immune defenses.

Notably, mucoid *A. baumannii* colonies are frequently isolated in clinical laboratories, and such mucoid strains are deemed to possess heightened virulence (Walker et al., 2019). Accumulating evidence has established that bacterial mucoidy constitutes an adaptive strategy to counteract environmental perturbations, and this phenotypic trait is closely associated with capsular thickness (Kon et al., 2020). Building on this finding, Shan et al. conducted additional investigations, which corroborated that *A. baumannii* with a mucoid phenotype exhibits markedly enhanced virulence (Shan et al., 2021). As a ubiquitous cell–cell communication system in bacteria, QS can regulate the formation of virulence-associated capsular polysaccharides and biofilms (Xie et al., 2019), implying its significant involvement in mucoidy and virulence regulation. Given the extensive regulatory influence of QS on bacterial community gene expression, investigating the relationship between QS, capsule production, and *A. baumannii* virulence is of paramount importance.

Previous QS research has predominantly employed standard strains, focusing on virulence phenotypes such as biofilm formation and bacterial motility. With the escalating prevalence of clinical carbapenem-resistant *A. baumannii* (CRAB) and mucoid strains, QS presents a significant clinical challenge. Therefore, investigating QS-mediated regulation of capsule production and host pathogenicity in clinical *A. baumannii* strains is of considerable significance. Building on previous studies indicating that interference with the QS signal molecule synthase gene *abaI* can attenuate *A. baumannii* virulence, the aim of this study was to investigate the effects of *abaI* on capsule production and virulence in *A. baumannii*, as well as its impact on the host immune response, using an *abaI* knockout strain generated via CRISPR/Cas9 technology in the clinical strain CRAB110.

## 2 Materials and methods

### 2.1 Materials

The mucoid *A. baumannii* strain CRAB110 was retrieved from the strain archive preserved at −80 °C in the laboratory of the School of Biology and Engineering (School of Modern Industry for Health and Medicine), Guizhou Medical University, Guiyang, China. This clinical isolate had been originally collected during routine diagnostic procedures at the Affiliated Hospital of Guizhou Medical University. As the bacterial isolate had been obtained for routine clinical purposes and was used retrospectively without involving identifiable patient information, the requirement for informed consent was waived by the Ethics Committee of Guizhou Medical University (approval No. [2023-394]). The *abaI* knockout strain (CRAB110Δ*abaI*) was constructed using CRAB110 as the parental strain. Additionally, the plasmids pSGAb and pCasAb were stored at −80 °C in the cryogenic freezer of the School of Biology and Engineering (School of Modern Industry for Health and Medicine), Guizhou Medical University. Strains CVO26 and KYC55 were acquired from Hangzhou Baosai Biotechnology Co., Ltd.

Specific pathogen-free (SPF) grade female BALB/c mice (6–8 weeks old) were purchased from Beijing Sibeifu Biotechnology Co., Ltd. (China). All animal experiments were performed in strict accordance with the Animal Welfare Guidelines and were approved by the Animal Ethics and Use Committee of Guizhou Medical University (Ethics Approval Number: 2302055).

The A549 and BEAS-2B cell lines employed in this study were preserved in the College of Biology and Engineering, Guizhou Medical University. Antibodies used included the rabbit monoclonal anti-NF-κB and p65 antibody (catalog Number: T55034) and rabbit monoclonal anti-GAPDH antibody (catalog Number: P60037F), both purchased from Abmart (Shanghai, China); the rabbit monoclonal anti-IL-17 antibody (Catalog Number: WL02981) from Wanleibio (Shenyang, China); and the goat anti-rabbit IgG-horseradish peroxidase (HRP)-conjugated secondary antibody from Shanghai Universal Biotech Co., Ltd.

### 2.2 Construction of *abaI* knockout strain

As previously described, the CRISPR/Cas9 method was used to construct the knockout strain (Wang et al., 2020). The spacer fragment was inserted into the pSGAb, and the reaction product was introduced by transformation into *Escherichia coli* DH5α competent cells for storage. The 40 nt DNA sequences upstream and downstream of the spacer sequence were selected as homologous repair template sequences. These sequences were spliced into a complete 80 nt homologous repair template sequence, *abaI*-ss DNA, which Biotech as a single-stranded DNA segment for *abaI* gene knockout. The pSGAb-*abaI* plasmid and ssDNA were co-electroporated into Ab-containing pCasAb, and colony PCR and sequencing were used to verify the gene knockout. The primers and plasmids used are listed in Supplementary Table S1.

### 2.3 Measurement of growth ability

Single colonies of the wild-type strain CRAB110 and the knockout strain CRAB110Δ*abaI* were picked and inoculated into 5 mL of LB broth (Harkova et al., 2024). They were incubated overnight at 37 °C with shaking at 100 rpm. The cells were subsequently collected by centrifugation, resuspended in sterile water to a 1.5 × 10^8^ CFU/mL, and subsequently used for growth curve experiments. The control group without inoculum received 200 μL of sterile LB medium. A 96-well plate was used to monitor the growth of each bacterial strain. The plate was incubated at 37 °C with shaking at 100 rpm, and the optical density (OD_600_) of the culture broth was measured every 4 h for a total of 24 h. The growth curve was then plotted based on these measurements. This experiment was conducted three times, with three replicate wells for each bacterial strain.

### 2.4 Measurement of surface motility

As described in the literature (Kim and Lee, 2016), motility plates were prepared by combining 5 g/L tryptone, 2.5 g/L NaCl, and 0.35% Eiken agar in ddH_2_O. The mixture was sterilized at 121 °C for 20 min and then poured into sterile petri dishes to prepare the agar plates. The plates were allowed to dry for approximately 2 h at room temperature in a biological safety cabinet. A 1-μL volume of bacterial suspension was pipetted into the center of each plate, with three replicates for each strain, and incubated at room temperature in a sterile environment for 30 min. Once the bacterial suspension was dry, the plates were placed in a constant-temperature incubator set at 30 °C, and the diameter of bacterial movement was measured after 24 h.

### 2.5 Detection of signal molecule content

The QS biosensor strain KYC55 was used to detect long-chain acyl-homoserine lactones (AHLs) signal molecules (Xu C. et al., 2024). KYC55 responds to exogenous AHLs by hydrolyzing X-gal and producing a color change. The presence of exogenous AHLs causes KYC55 to express the *lacZ* gene, resulting in the hydrolysis of X-gal and the appearance of a blue color. Signal molecule experiments were performed as described in previous studies (Tang et al., 2020). Firstly, 40 μL of X-gal (5-bromo-4-chloro-3-indolyl *β*-d-galactopyranoside, 20 mg/mL) was evenly spread across the surface of agar plates. After the plates were dried, *A. baumannii* and the sensor strain were inoculated side by side on the plates, maintaining a distance of at least 5 mm between them. The plates were incubated at 28 °C for 24 h, and color changes were observed.

### 2.6 Mucoid string test

Following the method described for the identification of mucoid bacteria in *Klebsiella pneumoniae* (Han et al., 2022; Kochan et al., 2023), bacteria were inoculated in a three-zone streak pattern on 5% sheep blood agar plates and incubated overnight at 37 °C. A bacterial inoculation loop was used to gently pick up and repeatedly stretch the *A. baumannii* colony on the plate. A positive mucoid string test was defined as a mucoid string length of at least 5 mm when the colony was picked up, and a negative result was defined as a mucoid string length less than 5 mm.

### 2.7 Congo red capsule staining and thickness measurement

The Congo Red-Crystal Violet staining technique can render the background of *A. baumannii* cells red, whereas the bacterial cells themselves appear dark purple, leaving the capsule unstained. The general procedure is as follows: The strain was revived as previously described, the Congo Red solution (0.5%) was mixed with fetal bovine serum in a 4:1 ratio, and then 10 μL of this mixture was added to a glass slide. A single colony from an overnight culture was mixed thoroughly with the liquid provided and allowed to dry. The mixed bacterial suspension was then decolorized with a 5% dilute hydrochloric acid solution for approximately 1 min. After decolorization, the slide was gently rinsed with running water until all residual color was removed. Subsequently, crystal violet staining solution was added to the bacteria mounted on the glass slide and stained for approximately 1 min. After staining, the slide was gently rinsed with running water to remove any excess crystal violet staining solution. The slide was then placed at room temperature to dry naturally. Once dry, the capsule of *A. baumannii* was examined under a microscope with an oil immersion lens. Observations were documented by taking photographs. The capsule thickness was measured using IFW software, with counts of 100 bacteria per strain (Shao et al., 2026).

### 2.8 Extraction and determination of capsular polysaccharides

First, the capsular polysaccharides were crudely extracted by spreading the wild strain CRAB110 and the knockout strain CRAB110Δ*abaI* on blood agar plates to reach conditions for capsular polysaccharide production. Subsequently, the bacteria from the surface of the plates were scraped off, nucleic acids were removed, and the capsular polysaccharides were precipitated out with ethanol (100%). In the second step, the content of the capsular polysaccharides was determined. Using a prepared glucose standard curve, a known quantity of the crude extracted capsular polysaccharides was weighed, and the absorbance was measured at a wavelength of 490 nm. After the colorimetric reaction was completed, the concentration of capsular polysaccharides in the sample was determined based on the standard curve (Zeng et al., 2022).

### 2.9 Monosaccharide composition

This experimental method was adapted from a previous study (Mao et al., 2024), with the brief procedure as follows: 5 mg of the STHP-5 sample was mixed with 1 mL of 2 mol/L trifluoroacetic acid (TFA) for hydrolysis at 121 °C for 2 h. After drying under a nitrogen atmosphere, the sample was washed with methanol and redried with nitrogen; this washing-drying cycle was repeated 3 times to remove TFA completely. The nitrogen-dried residue was then dissolved in 1 mL of deionized water, filtered through a 0.22 μm microporous membrane, and transferred to a chromatographic vial for high-performance anion-exchange chromatography (HPAEC) analysis. The analysis was performed on a Thermo ICS 5000+ system (Thermo Fisher Scientific, Waltham, MA, USA) equipped with a Dionex™ CarboPac™ PA-20 anion-exchange column (3 × 150 mm, 10 μm) and a pulsed amperometric detector (PAD). A 5 μL aliquot of the treated sample was injected. Gradient elution was conducted at 0.5 mL/min using mobile phases A (double-distilled water, ddH₂O), B (0.1 mol/L NaOH), and C (0.1 mol/L NaOH + 0.2 mol/L NaAc), with the elution program set as: 0 min (95:5:0, A:B:C), 26 min (85:5:10, held until 42 min), 42.1 min (60:0:40, held until 52 min), 52.1 min (95:5:0, held until 60 min). Experimental data were processed via the Chromeleon 7.2 Chromatography Data System (CDS), and the monosaccharide composition of STHP-5 was identified by comparing the sample’s retention time with that of the standard.

### 2.10 Density gradient experiment to determine the thickness of capsule

This method is based on the migration of bacterial cells through a gradient matrix due to centrifugal force, as detailed in this study (Kon et al., 2020). The gradient consists of multiple phases, each containing colloidal silica particles of 15–30 nm in diameter with different concentrations (20–80% V/V) of Percoll solution, purchased from Beijing Solarbio Science & Technology Co., Ltd. Bacterial cells are inoculated at the top of the gradient and, under centrifugal force, migrate to different positions within the density gradient based on their capsule size. A micro-gradient test is first conducted to determine the concentration that will provide the best separation. The density gradient medium is mixed with 1 × PBS to prepare a density gradient diluent. Aliquots of 500 μL of the single dilution are placed in 2 mL Eppendorf tubes for each micro-gradient test. The overnight cultures are centrifuged, and the pellets are collected and resuspended in 2 mL of 1 × PBS. Then, 100 μL of the bacterial suspension was added to the top of each gradient diluent and centrifuged at 8,000×*g* for 10 min in a microcentrifuge. After centrifugation, the tubes were transferred to a tube rack to observe the minimum gradient dilution required to retain each strain just above the gradient layer. Once the dilution was obtained where all strains could be retained just above the gradient, the formal experiment was conducted by transferring 500 μL of the least diluted density gradient solution into a 2-mL round-bottom EP tube, followed by the gentle addition of successively more concentrated gradient dilutions from below using a 1-mL syringe to avoid disruption of the layers. Finally, 200 μL of the bacterial suspension was added to the top layer and centrifuged at 3,000×*g* for 30 min. After centrifugation, the EP tubes were carefully removed and placed on an appropriate tube rack. The results were then documented with photographs.

### 2.11 Mouse survival rate experiment

Female BALB/c mice of SPF grade were purchased from Sibeifu (Beijing) Biotechnology Co., Ltd. Bacterial suspensions were prepared by centrifuging and collecting the overnight cultures of the wild-type strain CRAB110 and the knockout strain CRAB110Δ*abaI* that were grown to the exponential phase (Wang et al., 2024). The bacteria were washed with sterile saline 2 to 3 times and then adjusted to a concentration of 1 × 10^9^ CFU/mL for subsequent use. BALB/c mice, aged 6–8 weeks, were selected for survival analysis. The mice were randomly divided into three groups (*n* = 8 per group): the wild-type strain CRAB110 infection group, the CRAB110ΔabaI knockout strain infection group, and the sterile saline control group. After anaesthesia, 50 μL of bacterial suspension was injected via tracheal intubation to establish a mouse pulmonary infection model. The survival rates of the mice were recorded every 12 h from 1 to 7 days post-infection. After the experiment was completed, mice were humanely euthanized following institutional and national animal care guidelines. Before euthanasia, animals were fully anaesthetised with 5% isoflurane in oxygen until deep anaesthesia was confirmed by loss of reflexes. Cervical dislocation was then performed as the primary method, chosen for its rapid and humane action and in accordance with AVMA recommendations. The procedure was carried out only by trained personnel. Death was confirmed by the absence of respiration, heartbeat, and responses to stimuli. All procedures were approved by the Ethics Committee of Guizhou Medical University (Approval Number: 2302055).

### 2.12 Lung histopathological examination

After the injection of the bacterial suspension, mice were sacrificed at 4 h, 24 h, and 48 h, and lung tissues were collected. The tissues were fixed using a fixative solution containing 1 mL of paraformaldehyde per 1 g of tissue and were subsequently embedded in paraffin for sectioning. The sections were dewaxed, hydrated, stained with hematoxylin and eosin, and then scanned using a 3D panoramic scanner (model: 3D HISTECH Pannoramic 250, manufactured in Hungary). Histopathological observations were conducted, and the significant pathological changes within the sections were described in detail (Jackson-Litteken et al., 2025).

### 2.13 Lung edema

The lung edema in mice was evaluated by comparing the wet lung weight to the body weight of the mouse at 4 h, 24 h, and 48 h after the injection of the bacterial suspension (Zhang et al., 2026). The lung edema index was calculated using the following formula:

Lung edema index=(Wetlung weight[mg]/Body weight[g])×100%

### 2.14 Measurement of TNF-*α* and IL-6 in mouse serum

Orbital blood from mice was collected at 4 h, 24 h, and 48 h after the injection of the bacterial suspension. The sera were separated and stored at −80 °C. Subsequently, the levels of TNF-α and IL-6 were measured using an ELISA kit (Elabscience, E-EL-M3063, E-EL-M0044) (Pidaparthi et al., 2025; Gheorghiu et al., 2025).

### 2.15 Chemokine measurement

After 24 h of bacterial inoculation, tissue samples were taken from the left upper lung of the mice, and an appropriate amount of buffer was added to the tissue. Under ice bath conditions, the tissue was homogenized using a tissue homogenizer. Subsequently, the homogenate was incubated at 4 °C for 20 min and then centrifuged at 12,000 rpm for 10 min at 4 °C. The supernatant was collected and stored at −80 °C for further analysis. Quantitative detection of eight chemokines (CCL20, CXCL5, CCL24, CXCL10, CXCL11, CCL12, CCL19, CCL27), was performed using Luminex technology (Wang et al., 2025).

### 2.16 Adhesion of A549 and BEAS-2B cells

A549 lung carcinoma epithelial cells and BEAS-2B normal human bronchial epithelial cells were cultured in DMEM supplemented with 10% fetal bovine serum (FBS), 100 μg/L streptomycin, and 100 U/mL penicillin at 37 °C in a humidified atmosphere containing 5% CO_2_. Cells were seeded into 24-well plates and cultured to reach approximately 90% confluence for the subsequent adhesion experiments. Before infection, the A549 and BEAS-2B cells were washed 2–3 times with sterile PBS, and the culture medium was replaced with pure DMEM devoid of serum or antibiotics. Bacterial adhesion assay: Bacteria (1 × 10 ^6^ CFU) were added to monolayers of A549 and BEAS-2B cells and co-cultured for 2 h at 37 °C. After infection, the cells were washed 3 times with PBS, lysed in 1 mL of Triton X-100, and the lysates were then plated onto LB agar plates. Colony counts were determined after an overnight incubation at 37 °C to assess the number of bacteria adhering to A549 and BEAS-2B cells (Liu et al., 2022).

### 2.17 RAW264.7 phagocytosis assay

Mouse-derived macrophages (RAW264.7) were cultured in RPMI 1,640 medium supplemented with 10% fetal bovine serum (FBS), 100 μg/mL streptomycin, and 100 U/mL penicillin, at 37 °C and 5% CO_2_. For the experiment, 200 ng/mL lipopolysaccharide (LPS) was used to induce macrophage polarization. The cells were seeded in 24-well plates to reach 90% confluence before experimental assays. Before infection, A549 cells were washed three times with sterile PBS. The culture medium was then replaced with RPMI 1,640 medium, which was devoid of serum and antibiotics. Bacteria (1 × 10^7^ CFU) were added, and the co-culture was incubated at 37 °C for 2 h. After infection, the cells were washed three times with PBS, treated with 500 μL of gentamicin (100 μg/mL) for 1 h to kill adherent bacteria, washed twice with PBS, and then lysed with 1 mL of Triton X-100. The lysates were plated onto LB agar plates and incubated at 37 °C overnight. The resulting colonies were counted to estimate the number of bacteria phagocytosed by the macrophages (Zhu et al., 2024).

### 2.18 Western-blot

Proteins from the lung cancer epithelial cell line A549 were extracted by sonication following lysis with a lysis buffer. Protein concentrations were determined using the BCA Protein Assay Kit. The protein samples were then stored at −20 °C. SDS-PAGE gels were prepared, and the samples were loaded. Proteins were separated by electrophoresis, and transferred to PVDF membranes. The membrane was blocked with 5% non-fat dry milk for 1 h. It was then incubated overnight at 4 °C on a shaker with the primary antibody (I), washed three times with TBST, and subsequently incubated with the corresponding secondary antibody (II) for 1 h at room temperature. Afterward, the membrane was rewashed three times with TBST and visualized using an ECL chemiluminescent reagent with a gel imaging system (Yang et al., 2024). GAPDH served as the internal control, and the protein bands were analyzed using ImageJ software.

### 2.19 Prokaryotic transcriptomic analysis

High-throughput sequencing technology was employed to sequence the transcriptome analysis of *A. baumannii*, aiming to analyze and compare the differential expression of transcripts between the wild-type strain CRAB110 and the knockout strain CRAB110Δ*abaI*. The procedure was as follows: single colonies were inoculated into LB liquid medium and cultured at 37 °C with shaking at 220 rpm until the logarithmic growth phase overnight. Subsequently, the bacteria were centrifuged to collect the cell pellets, with three biological replicates for each group, resulting in a total of six samples. The collected bacterial pellets were immediately frozen in liquid nitrogen and stored at −80 °C before being sent to Shanghai Meiji Bio-pharmaceutical Technology Co., Ltd. for library construction and sequencing analysis (Cirino et al., 2023). Sequencing was carried out on the Illumina platform, and the resulting clean data were aligned with the reference genome ATCC17978. After quantifying gene expression, differentially expressed genes were identified using a cutoff of log2 (fold change) ≥ 1 and a *p*-adjusted value <0.05. The RNA-seq data obtained in this study have been submitted to SRA with the accession number PRJNA1143159.

### 2.20 Lung transcriptomics analysis

The wild strain CRAB110 and knockout strain CRAB110Δ*abaI*, which were cultured overnight to reach the logarithmic growth phase, were handled separately. The concentration of the bacterial suspension and the infection method were consistent with previous studies. Twenty-four hours post-infection, the left upper lobe of the mouse lung was excised according to the standard protocol, rinsed with sterile RNase-free water, transferred to a cryotube, flash-frozen in liquid nitrogen, and stored at −80 °C. Subsequently, the tissue was sent to Beijing Novogene Technology Co., Ltd. for library construction and sequencing analysis (Wang et al., 2019). The RNA-seq data obtained in this study have been submitted to SRA with the accession number PRJNA1145176.

### 2.21 qRT-PCR validation

#### 2.21.1 qRT-PCR validation of prokaryotic transcriptome

The expression levels of five genes: *wzb* (low molecular weight protein tyrosine phosphatase, HKO16_RS18735), the gene encoding a DDE-type integrase/transposase/recombinase (HKO16_RS17835), the gene encoding a zeta toxin family protein (HKO16_RS17870), *xth* (exodeoxyribonuclease III, HKO16_RS17575), and the gene encoding a non-ribosomal peptide synthetase (*NRPS*; HKO16_RS13995)—were detected by quantitative real-time PCR (qRT-PCR) to validate the RNA-Seq results.

Bacterial samples were prepared, and total RNA was extracted using the M5 EASY spin plus kit (Mei5 Biotechnology Co., Ltd., China). Then, 1 μg of RNA was reverse-transcribed into cDNA using the PrimeScript RT reagent kit. The qRT-PCR reactions were set up according to the manufacturer’s instructions provided with the SYBR Premix Ex Taq kit. Gene expression levels were normalized to the 16S rRNA gene, and relative expression levels were calculated by the 2^-ΔΔCT^ method (Livak and Schmittgen, 2001). The primers used are listed in Supplementary Table S2.

#### 2.21.2 qRT-PCR validation of mouse lung transcriptome

The expression levels of five genes (TNF, IL-6, TNFAIP3, IL-1β, CCL4) were assessed by quantitative reverse transcription PCR (qRT-PCR) to validate the RNA-Seq findings. Tissue samples were processed for transcriptomic analysis, and total RNA was isolated using the Trizol reagent method. Subsequently, 1 μg of RNA was converted to cDNA by reverse transcription with the PrimeScript RT Kit. The reaction mixture was prepared as per the manufacturer’s instructions for the SYBR Premix Ex Taq kit. Glyceraldehyde-3-phosphate dehydrogenase (GAPDH) served as the internal control gene, and relative expression levels were determined using the 2^-ΔΔCT^ method (Livak and Schmittgen, 2001). The sequences of the primers used are provided in Supplementary Table S3.

### 2.22 Immune cell infiltration analysis

The abundance of immune cells in the mouse transcriptome was quantified using the R package CIBERSORT (version 0.1.0). A total of 22 immune cell types were identified, including 7 T cell subtypes (CD8^+^ T cells, Naive CD4^+^ T cells, Resting memory CD4^+^ T cells, Activated memory CD4^+^ T cells, Follicular helper T cells, Regulatory T cells, Gamma delta T cells), Naive B cells, Memory B cells, Plasma cells, NK cells (including Resting NK cells and Activated NK cells), and bone marrow-derived immune cells (such as Monocytes, M0 Macrophages, M1 Macrophages, M2 Macrophages, Resting dendritic cells, Activated Dendritic cells, Resting mast cells, Activated mast cells, Eosinophils, and Neutrophils) (Chen et al., 2018; Newman et al., 2015). The results were visualized using box plots generated with the “ggplot2” package.

### 2.23 Statistical analysis

All data are presented as the mean ± standard deviation (SD). Statistical analysis and graphing were performed using GraphPad Prism software (version 10.1.2, GraphPad Software, Boston, MA, USA). Comparisons between two groups were analyzed using Student’s two-tailed unpaired t-test. A *p* value < 0.05 was considered statistically significant.

## 3 Results

### 3.1 Effect of *abaI* knockout on virulence-related phenotypes

#### 3.1.1 Faster growth ability of the strain after *abaI* gene knockout

**Figure 1:**
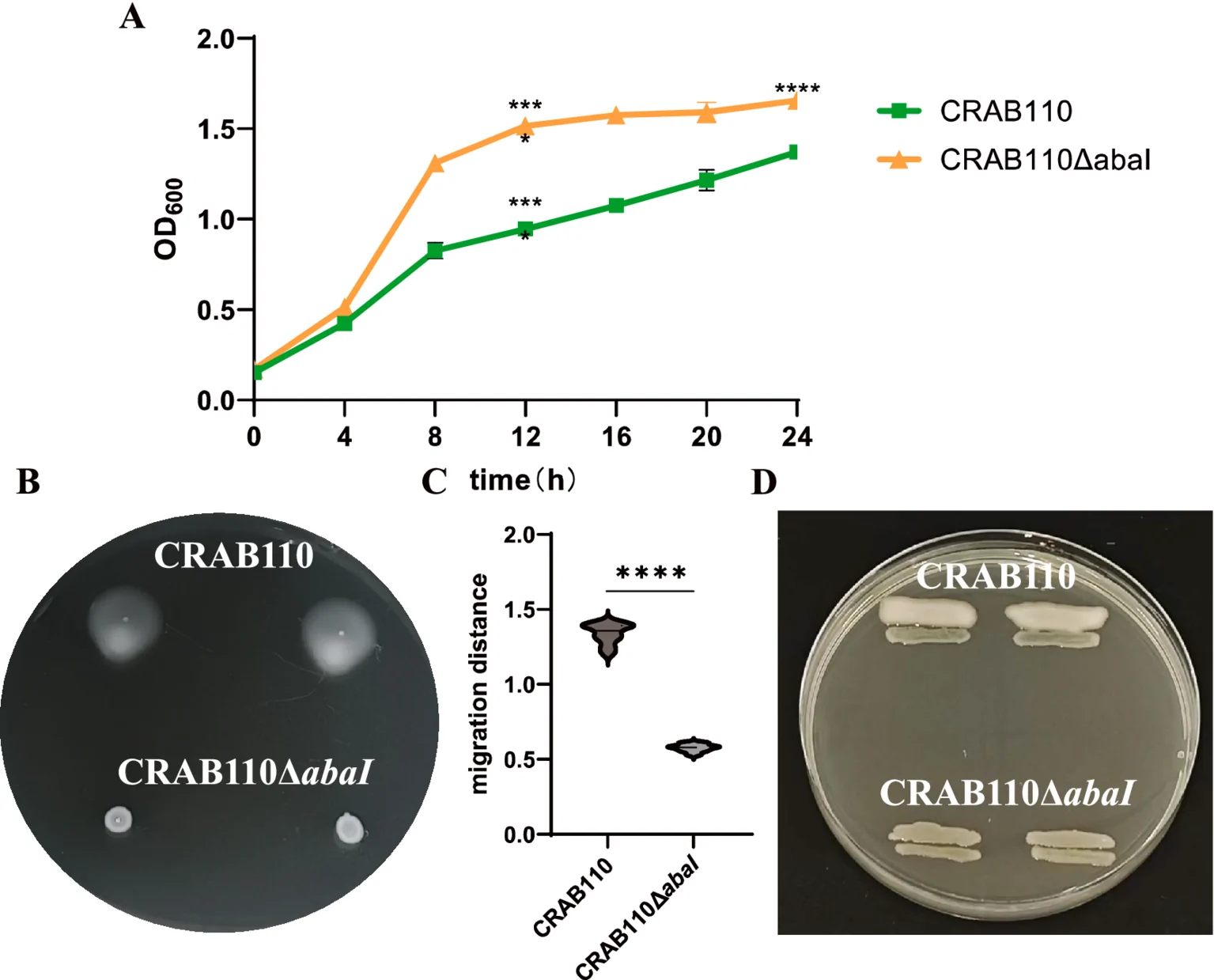
Measurement of growth ability, motility, and quorum sensing signal molecules. **(A)** Bacterial growth curves. **(B)** Determination of surface motility. The strain was inoculated onto the surface of a semi-solid agarose plate at 0.35% concentration and incubated at 30 °C for 24 h. Compared with the CRAB110 and the CRAB110Δ*abaI* exhibited defects in surface-associated motility. **(C)** Statistical graph of the migration distance of CRAB110 and CRAB110Δ*abaI*. **(D)** Color reaction assays were performed on the wild-type, knockout, and CVO26. ^***^p < 0.001.

The knockout strain CRAB110Δ*abaI* was successfully constructed using the CRISPR/cas9 system (Supplementary Figure S1). To investigate the role of the *abaI* gene in the growth curve of *A. baumannii*, we monitored the OD_600_ of the cultures over time (Figure 1A). Compared with the CRAB110, the CRAB110Δ*abaI* showed enhanced growth during the exponential phase.

#### 3.1.2 Reduced surface motility after *abaI* gene knockout

To investigate the effect of the QS gene *abaI* on the motility of *A. baumannii*, we conducted a motility plate assay to measure the motility by assessing the diameter of the strain’s migration (Figure 1B). The knockout strain CRAB110Δ*abaI* showed significantly reduced motility compared with the wild-type strain CRAB110 (Figure 1C). The results suggest that the *abaI* gene contributes to bacterial motility.

#### 3.1.3 No production of signaling molecules after *abaI* gene knockout

To explore the effect of the QS gene *abaI* on AHL (acyl-homoserine lactone) production in the strain, we used the KYC55 strain for long-chain signaling molecules. However, the wild-type strain CRAB110 elicited a color reaction with KYC55, indicating that the CRAB110 produced long-chain signaling molecules. In contrast, the CRAB110Δ*abaI* strain did not show a color reaction (Figure 1D). The result suggests that the deletion of the *abaI* gene affects the secretion of signaling molecules.

#### 3.1.4 Altered strain morphology after *abaI* gene knockout

**Figure 2:**
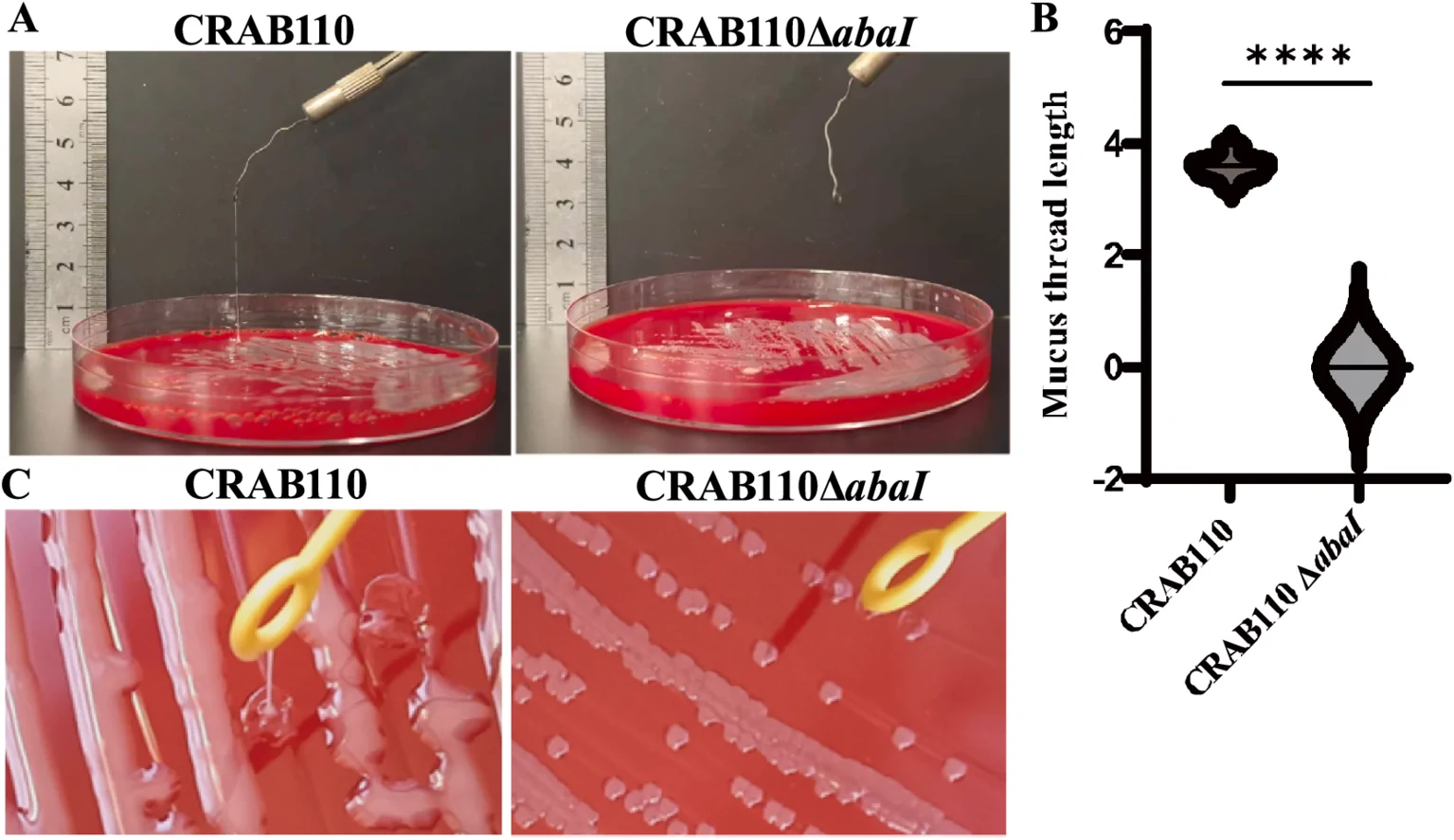
Effect of *abaI* knockout on bacterial capsule production. **(A)** Bacteria were inoculated onto blood agar plates using an inoculating loop for the mucus-pulling assay; **(B)** Statistical analysis diagram of bacterial mucin stringing assay. **(C)** Effects of *abaI* gene knockout on colony phenotype.

The role of the *abaI* gene was explored by analyzing changes in mucilage production, capsule production, thickness, and morphology after the *abaI* gene knockout. The results showed that the wild strain CRAB110 was positive for mucilage production (Figure 2A), and the length of mucus thread can reach up to 4 cm, whereas the mucoid colonies of CRAB110Δ*abaI* were negative (Figure 2B). Colony phenotype observation showed that CRAB110 was smooth and moist, whereas CRAB110Δ*abaI* displayed a rough and dry appearance (Figure 2C). These results suggest that the *abaI* gene affects the mucilage production.

#### 3.1.5 *abaI* gene knockout weakened the capsule production of the strain

**Figure 3:**
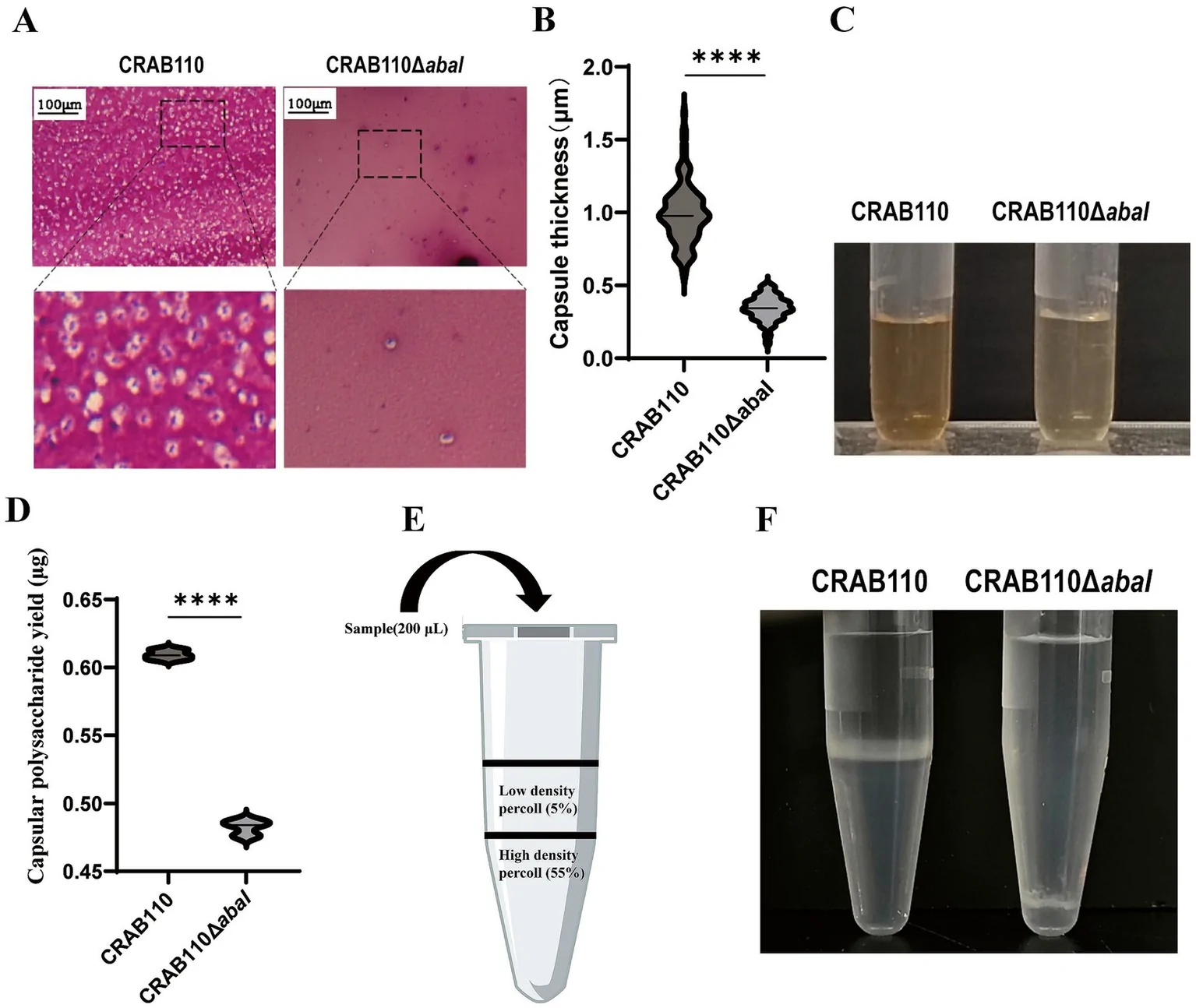
Effect of *abaI* gene knockout on bacterial capsule. **(A)** Capsule staining. The background and bacteria were stained negatively, with the bacteria appearing purple and the capsule remaining colorless around them. **(B)** Capsule thickness measurement. The thickness of the stained capsule was measured using IFW software, with results showing ^***^p < 0.001. **(C)** Extraction of capsular polysaccharide. The phenol-concentrated sulfuric acid method was used to extract polysaccharide, and absorbance detection was performed at 490 nm. **(D)** Statistical chart of capsular polysaccharide content. **(E)** Bacterial cells are added to the top of a gradient medium consisting of two layers (5 and 55% Percoll separation solution) in the manner shown in the figure. **(F)** After centrifugation, the bacterial cells migrate to the top phase of the gradient. Those strains above the line may possess a capsule, while those below the line indicate that the strain is either thin or capsule-free.

The capsule is a mucus-like substance produced by bacteria around their cell walls throughout their life cycle. It is composed of polysaccharides and aids in the colonization of bacteria in their hosts. Since the formation of mucoid colonies in strains is often associated with capsule formation, a series of experiments on capsule formation was conducted to further explore the relationship between the *abaI* gene and the strain’s capsule. Initial capsule staining experiments revealed that the wild strain CRAB110 had a thicker capsule, whereas the knockout strain exhibited a significantly reduced capsule thickness compared to the wild type (Figures 3A,B). Capsule polysaccharides are one of the virulence factors, and variations in capsule thickness due to knockout and complementation in different strains may result in different virulence outcomes. Furthermore, crude extraction of capsule polysaccharides was performed for both the wild-type strain CRAB110 and the knockout strain CRAB110Δ*abaI*. The polysaccharide concentration was determined using the phenol-sulfuric acid method (Figure 3C). The concentration of the sample in CRAB110 was 77.24 μg/mL, whereas the concentration in CRAB110Δ*abaI* was 11.85 μg/mL. These findings suggest that the polysaccharide content of CRAB110Δ*abaI* is lower than that in the CRAB110.

Density gradient experiment, a method commonly used to assess the thickness of bacterial capsule, was carried out by suspending bacterial cells at varying heights within a gradient through dilution and centrifugation. Preliminary gradient experiments were conducted initially to identify suitable gradient concentrations (5, 55%) capable of suspending both viscous and less viscous strains for subsequent formal experiments (Figure 3D). The results showed that CRAB110 was located at higher positions within the separation medium, but the knockout strain at a lower level. This indicates that the capsule of CRAB110 is stronger than CRAB110Δ*abaI* (Figure 3E). Based on the above results, this indicates that deletion of the *abaI* gene alters the morphology and viscosity of the strain, significantly reducing its capacity to produce a capsule.

#### 3.1.6 Knockout of the *abaI* gene reduces the galactose component in the capsule

In this study, the capsular polysaccharide components of CRAB110 and CRAB110Δ*abaI* were analyzed. The results showed that among these polysaccharide components, the content of galactose, fructose, and glucuronic acid significantly decreased after *abaI* gene knockout (Table 1).

**Table 1:** Effect of *abaI* gene knockout on capsular polysaccharide components.

Composition	CRAB110	CRAB110Δ*abaI*
Fuc (μg/mg)	1.40 ± 0.059 a	0.00 ± 0.000 b
Ara (μg/mg)	0.00 ± 0.000 a	0.00 ± 0.000 a
Rha (μg/mg)	0.00 ± 0.00 a	0.00 ± 0.000 a
Gal (μg/mg)	5.27 ± 0.090 a	1.48 ± 0.051 b
Glc (μg/mg)	1.29 ± 0.017 b	2.57 ± 0.159 a
Xyl (μg/mg)	0.00 ± 0.000 a	0.00 ± 0.000 a
Man (μg/mg)	0.00 ± 0.000 a	0.00 ± 0.000 a
Fru (μg/mg)	0.00 ± 0.000 a	0.00 ± 0.000 a
Rib (μg/mg)	0.81 ± 0.063 b	7.55 ± 0.090 a
Gal-UA (μg/mg)	0.00 ± 0.000 a	0.00 ± 0.000 a
Gul-UA (μg/mg)	0.00 ± 0.000 a	0.00 ± 0.000 a
Glc-UA (μg/mg)	7.55 ± 0.100 a	0.00 ± 0.000 b
Man-UA (μg/mg)	0.00 ± 0.000 a	0.00 ± 0.000 a

Lowercase letters indicate the significance of the same indicator among different groups. Identical letters mean there is no significant difference between the two groups, while other letters indicate a significant difference between the two groups.

### 3.2 *abaI* gene knockout weakened the pathogenicity of the strain

#### 3.2.1 The survival rate of mice increased after *abaI* gene knockout

**Figure 4:**
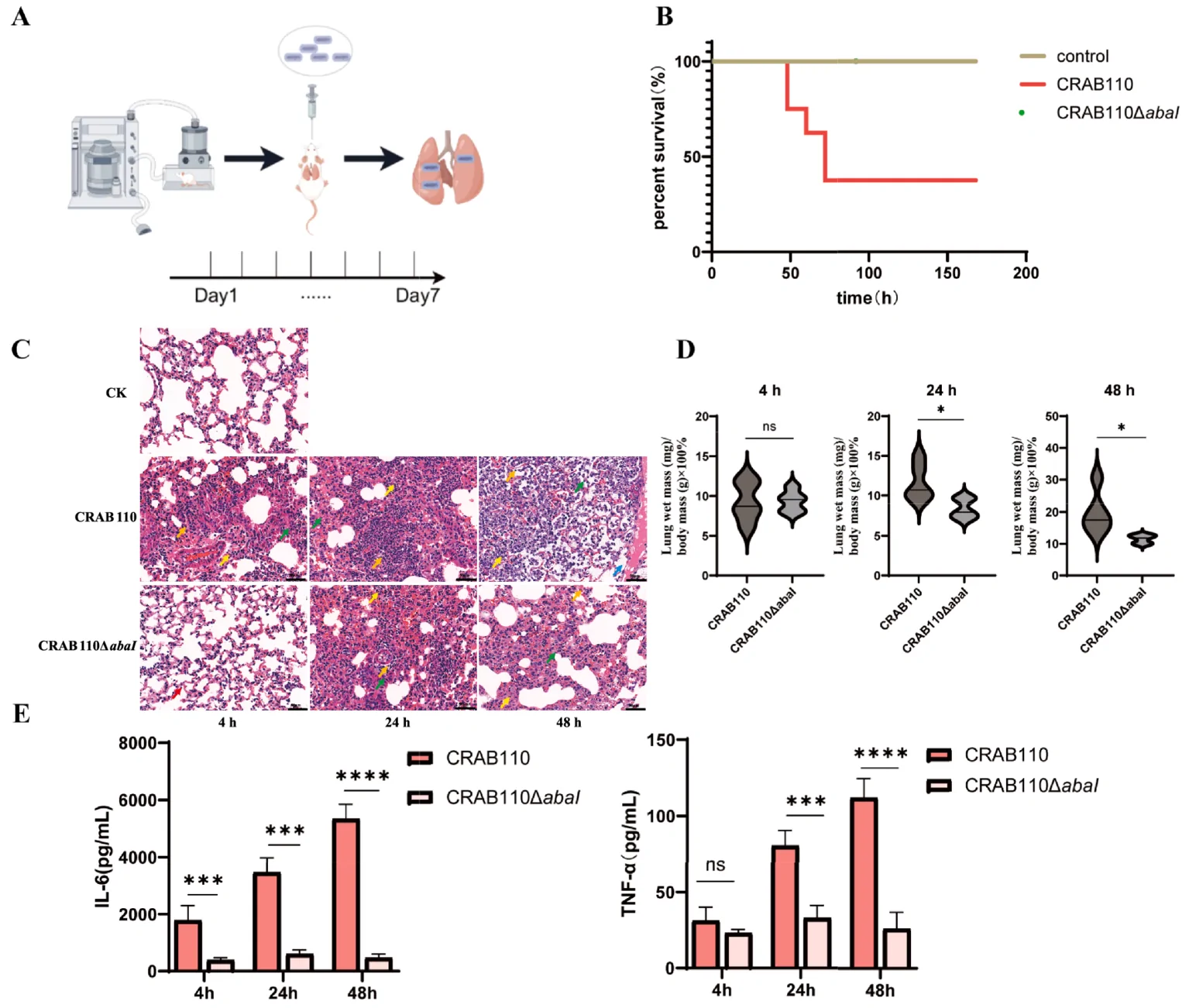
Survival and pulmonary inflammation in infected mice. **(A)** Schematic diagram of the construction of the mouse pneumonia model. The diagram was drawn using Figdraw (www.figdraw.com). **(B)** Mouse survival rate experiment (*n* = 8). **(C)** Pathological sections of the lungs from strain-infected mice at 4, 24, and 48 h. The magnification is 400 times, and the scale bar represents 50 μm. Locations indicated by differently colored arrows in the figure correspond to distinct cells or bacteria: Alveolar epithelial cell necrosis (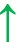), lymphocytes (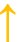), neutrophils (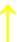), desquamation of alveolar epithelial cells (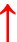), bacteria (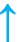). **(D)** Lung edema in strain-infected mice at 4, 24, and 48 h (*n* = 5). **(E)** ELISA determination of serum IL-6 and TNF-*α* in strain-infected mice at 4, 24, and 48 h. ^*^p < 0.05, ^***^p < 0.001, *ns* indicates no statistical significance.

To evaluate the impact of the *abaI* gene on the virulence of *A. baumannii*, we constructed a mouse lung infection model and used this model to assess the survival rate of mice after infection. Mice received intratracheal instillation of bacterial suspension and were monitored over a 7-day period. They were then placed in a closed space with isoflurane gas, and subsequently, the bacterial solution was delivered to the lungs of the mice using a butterfly tracheal intubation needle (Figure 4A). Mice injected with the wild strain CRAB110 began to die at 48 h, with a total of 5 deaths by 72 h. Mice injected with the knockout strain CRAB110Δ*abaI* showed no deaths throughout the 7-day observation period (Figure 4B). These results indicate that the wild-type strain CRAB110 has high virulence, which is reduced after the knockout of the *abaI* gene.

#### 3.2.2 *abaI* gene knockout weakened the inflammatory infiltration

Histopathological examination of the left inferior lung lobe in mice was performed using hematoxylin and eosin (HE) staining at 4, 24, and 48 h after bacterial challenge (Figure 4C). At 4 h post-infection, lung tissues infected with the wild-type strain CRAB110 showed serosal covering without significant edema, inflammation, or fibrosis. Bronchial structures remained intact with normal epithelial morphology. Alveolar epithelial cell necrosis was observed, featuring nuclear shrinkage and interstitial infiltration of lymphocytes and neutrophils. Similarly, tissues infected with the knockout strain CRAB110Δ*abaI* exhibited no notable pathology beyond minor alveolar cell shedding and absence of interstitial inflammation. By 24 h, all groups showed increased inflammatory infiltration, most severe with CRAB110. At 48 h, CRAB110 infection resulted in marked alveolar epithelial necrosis, cytoplasmic dissolution, nuclear pyknosis, and cell shedding, accompanied by interstitial lymphocytic and neutrophilic infiltration. In contrast, inflammatory levels in the knockout strain groups decreased relative to 24 h, suggesting partial immune-mediated clearance of these strains.

#### 3.2.3 *abaI* gene knockout strain weakened the pulmonary edema in mice

After lung modeling in mice, there was no significant difference in the weight of lung tissue among the three groups at 4 h post-infection. As time progressed, all groups of mice developed varying degrees of lung edema. Mice infected with the wild-type strain CRAB110 exhibited the most severe lung edema. Notably, at 24 and 48 h post-infection, lung edema was significantly lower in mice infected with the CRAB110Δ*abaI* mutant strain compared with those infected with the wild-type strain CRAB110 (*p* < 0.05, Figure 4D).

#### 3.2.4 *abaI* gene knockout reduces the secretion level of TNF-*α* and IL-6 in infected mice

TNF-α and IL-6 are essential pro-inflammatory cytokines in the host. To determine the secretion levels of these cytokines following infection, we detected TNF-α and IL-6 in the sera of infected mice. The results showed that at 4 h post-infection, there was no significant difference in TNF-α levels between the sera of mice infected with the knockout strain CRAB110Δ*abaI* and the wild-type strain CRAB110. However, the level of IL-6 in the sera of mice infected with the CRAB110Δ*abaI* was significantly lower compared to those in the CRAB110. At 24 h, the levels of TNF-α and IL-6 increased in the sera of mice infected with CRAB110 and CRAB110Δ*abaI*. Compared to the CRAB110, the levels of TNF-α and IL-6 were significantly reduced in the sera of mice infected with the knockout strain CRAB110Δ*abaI*. At 48 h, the levels of TNF-α and IL-6 continued to elevate in the sera of mice infected with CRAB110 and CRAB110Δ*abaI*. Yet, the levels in mice infected with the knockout strain remained significantly lower than those in mice infected with the wild-type strain CRAB110 (Figure 4E).

#### 3.2.5 *abaI* mediates the expression of key host chemokines

**Figure 5:**
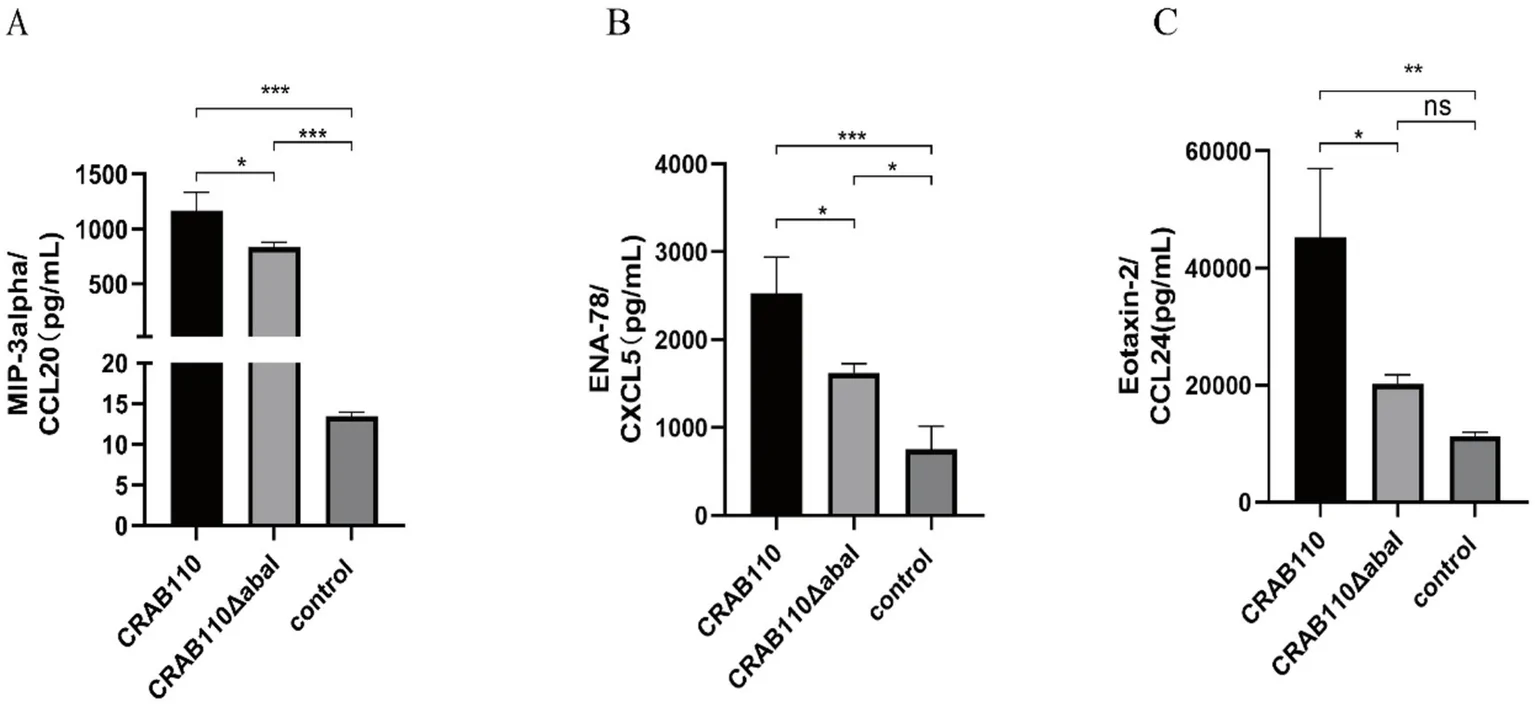
The impact of *abaI* gene knockout on host chemokine expression. **(A–C)** Determination of chemokines of CCL20, CXCL5, and CCL24. The control group consisted of lung tissue from mice injected with saline. ^*^p < 0.05, ^**^p < 0.01, ^***^p < 0.001; ns indicates no statistical significance.

Cellular immunity is crucial for the host’s immune response in clearing pathogens, and chemokines CCL20, CXCL5, CXCL10, and CCL24 are all critical immune molecules that play an essential role in resistance to external aggression. To assess the levels of these cytokines in the lungs of infected mice, we performed assays on lung tissue samples. The results showed that the chemokines CCL20, CXCL5, and CCL24 all showed a trend of expression levels with CRAB110 > CRAB110Δ*abaI* > control group (Figures 5A–C); however, the expression level of CCL24 was not significantly different at either the strong or weak level between the CRAB110Δ*abaI* and control groups (Figure 5C). The chemokines CXCL10 and CXCL11 demonstrated a trend of expression levels with CRAB110Δ*abaI* > CRAB110 > control, but this trend was not statistically significant between the CRAB110 and control groups (Supplementary Figures S2A,B). The expression levels of the chemokine CCL12 in both CRAB110 and CRAB110Δ*abaI* were not statistically significant, yet both were significantly different from the control group in comparison with CRAB110 (Supplementary Figure S2C). The expression levels of CCL19 and CCL27 did not differ significantly among the three groups (Supplementary Figures S2D,E). Taken together, these findings suggest that both the CRAB110 wild-type strain and the CRAB110Δ*abaI* knockout strain induced some level of host infection when injected into the lungs of mice for 24 h. Compared to the CRAB110 wild-type strain, the reduced expression of chemokines CCL20, CXCL5, and CCL24 in the CRAB110Δ*abaI* knockout strain may be one of the reasons for the reduced pathogenicity of the knockout strain in mice infected with CRAB110Δ*abaI*.

#### 3.2.6 *abaI* gene knockout enhances host adhesion and phagocytosis

By selecting lung-derived cell lines A549 and BEAS-2B, the adhesion of bacteria to lung epithelial cells was simulated, and the effect of *abaI* knockout on bacterial adhesion to host cells was investigated. The results of strain adhesion to the epithelial cell lines A549 and BEAS-2B (Supplementary Figures S3A,B), indicate that the knockout strain CRAB110Δ*abaI* exhibited stronger adhesion compared to the wild-type strain CRAB110, with a statistically significant difference. The presence of CPS can impair complement-mediated bacterial killing and phagocytosis by neutrophils and macrophages. An experiment on the phagocytosis of RAW264.7 macrophages was conducted to assess the effect of the *abaI* knockout on the phagocytosis rate of immune cells. The phagocytosis data revealed that the knockout strain CRAB110Δ*abaI* was ingested by murine RAW264.7 macrophages in significantly higher numbers than the wild-type strain CRAB110 (Supplementary Figure S3C), suggesting that the wild-type strain CRAB110, with its thick capsule, can evade the immune response during host infection, thereby facilitating the strain’s ability to infect and cause disease in the host. In contrast, the knockout strain CRAB110Δ*abaI*, with a thinner capsule, was more efficiently phagocytosed by macrophages, which may reduce its infectivity and pathogenicity in the host.

#### 3.2.7 *abaI* gene activates inflammation of pulmonary epithelial cells infected by *A. baumannii* myxoid through IL-17 and NF-κB signaling pathways

**Figure 6:**
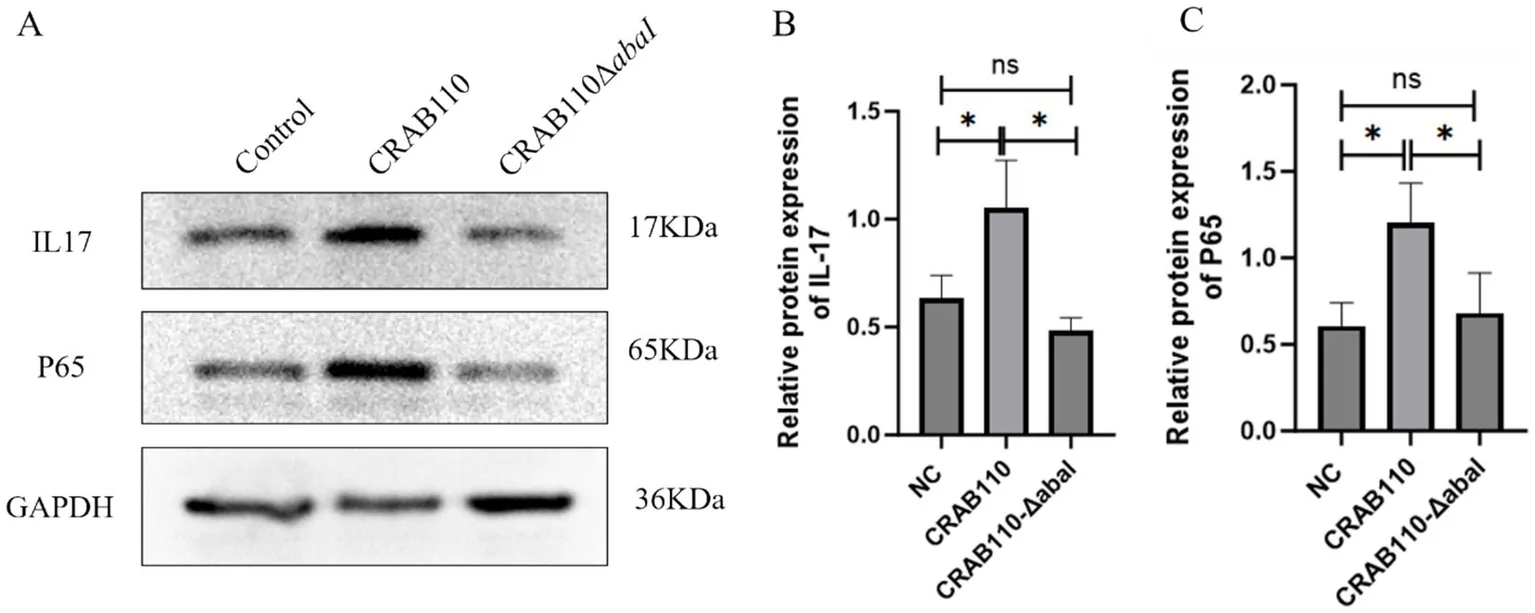
IL-17 and p65 protein expression in lung epithelial cells A549. **(A)** Western blot analysis of IL-17 and p65 expression in A549 cells. **(B)** The relative expression level of IL-17 protein in each group. **(C)** The relative expression level of p65 protein in each group. ^*^p < 0.05, ^**^p < 0.01, and ns is not statistically significant.

To verify whether the *abaI* gene activates the inflammatory response in pulmonary epithelial cells infected with *A. baumannii* through the IL-17 and NF-κB signaling pathways, we measured the expression of proteins related to these pathways. The results indicated that lung epithelial cells infected with the CRAB110 strain exhibited a significant increase in the level of IL-17-related proteins compared to the uninfected group. Moreover, the level of IL-17 protein in lung epithelial cells infected with the CRAB110Δ*abaI* strain was significantly reduced compared with the CRAB110. These results suggest that the *abaI* gene could activate the IL-17 signaling pathway (Figures 6A,B; Supplementary Figure S4). Furthermore, we evaluated the expression level of NF-κB p65 within the NF-κB signaling pathway. We observed a notable increase in p65 protein expression in the CRAB110-infected group, as compared to the uninfected control group (Figures 6A,C; Supplementary Figure S4). Additionally, lung epithelial cells infected with the *abaI* knockout strain exhibited significantly lower levels of p65 protein compared to those infected with wild-type strains, indicating that *abaI* plays a crucial role in promoting the activation of the NF-κB inflammatory pathway.

### 3.3 Molecular mechanism of decreased virulence after *abaI* gene knockout

#### 3.3.1 Transcriptome analysis of knockout strain

**Figure 7:**
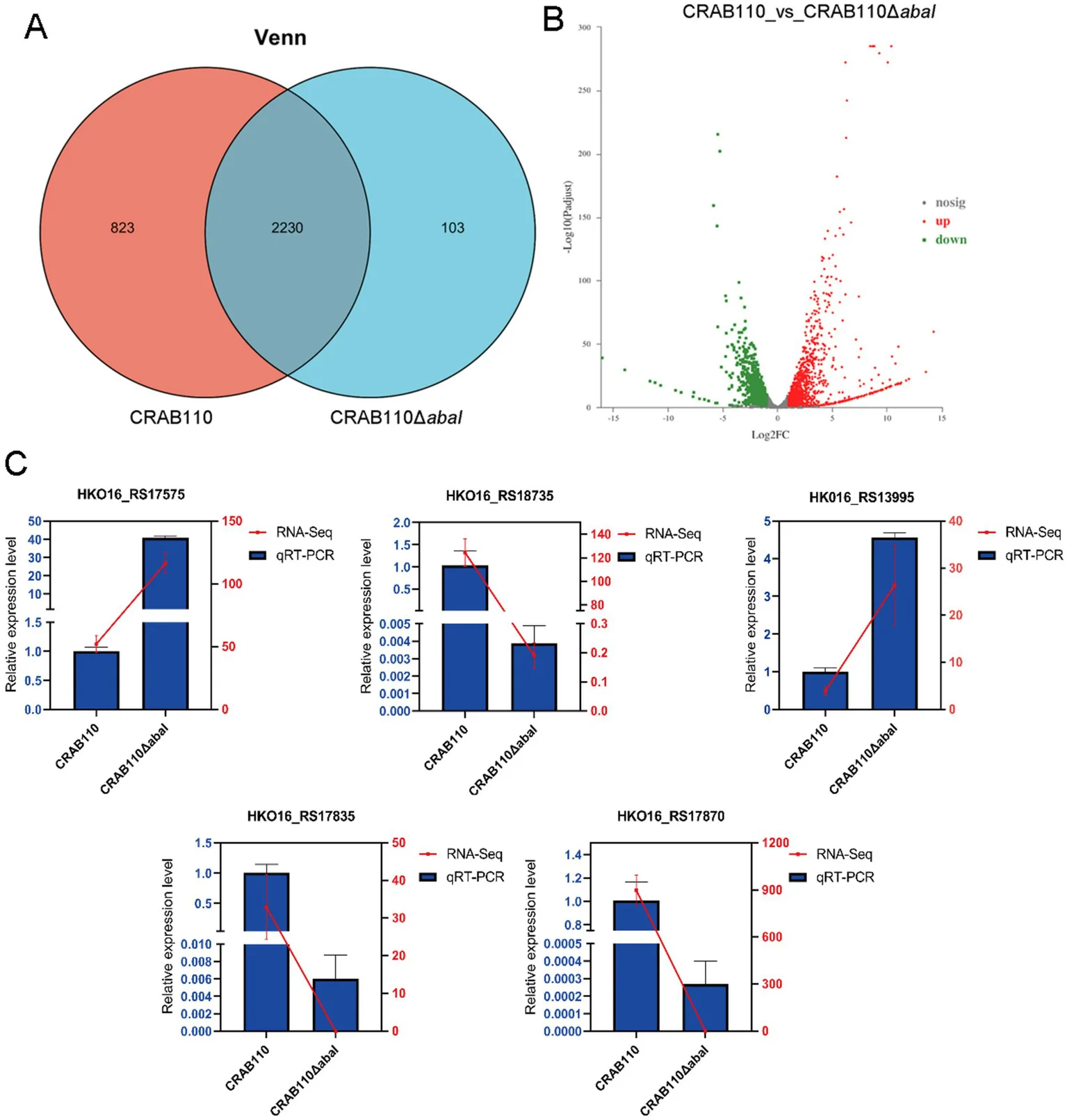
Analysis of prokaryotic transcriptome expression levels and qRT-PCR validation. **(A)** Venn diagram of gene expression levels. The orange circle represents genes annotated in the wild strain, whereas the blue circle represents genes annotated in the knockout strain. The overlapping area in the center indicates genes commonly annotated in both bacterial strains. **(B)** Volcano plot of differentially expressed genes. Red indicates up-regulated genes, green indicates down-regulated genes, and gray represents genes with no significant difference. **(C)** qRT-PCR validation. This figure shows the relative expression levels of five differentially expressed genes. The results are presented as the mean ± standard deviation from at least three biological replicates.

Transcriptome samples were analyzed for both the wild-type strain CRAB110 and the knockout strain CRAB110Δ*abaI*. Gene expression analysis indicated that 3,053 genes were expressed in the wild-type strain and 2,333 genes in the knockout strain, with 2,230 genes co-expressed in both strains (Figure 7A). For these co-expressed genes, applying a screening threshold of |log2FC| ≥ 1 and *P*-adjust < 0.05, a total of 1,772 differentially expressed genes (DEGs) were identified, including 984 genes that were up-regulated and 788 genes that were down-regulated (Figure 7B). Genes related to capsule production with the most significant differences, such as *wzb* (HKO16_RS18735), the virulence-related genes *HKO16_RS17835* and *HKO16_RS17870*, as well as other genes with significant differences, *HKO16_RS17575* and *HKO16_RS13995*, were validated by qRT-PCR (Figure 7C). The results confirmed that the expression trends of the differentially expressed genes agreed with the high-throughput sequencing data, indicating the reliability of the sequencing quality.

Through GO and KEGG analyses of these differentially expressed genes, the results revealed that after knocking out the *abaI* gene, GO enrichment identified a total of 129 pathways, with the bubble chart depicting only the top 20 most significantly altered pathways (Supplementary Figure S5A). Among these, biological processes encompassed 80 pathways, including those involved in the carbohydrate derivative metabolic process, tRNA metabolic process, and monosaccharide metabolic process. Cellular components were enriched in 14 pathways, specifically the membrane protein complex, plasma membrane protein complex, transmembrane transport complex, transporter complex, ATP-binding cassette (ABC) transporter complex, and ATPase-dependent transmembrane transport complex. Molecular functions included activities such as catalysis acting on tRNA, intramolecular oxidoreductase activity, aldose and ketose interconversion, and aminoacyl-tRNA ligase activity. The results of the KEGG pathway enrichment analysis showed that these differentially expressed genes were primarily enriched in 6 pathways, including aminoacyl-tRNA biosynthesis, Ribosome, glycolysis/gluconeogenesis, sulfur metabolism, the pentose phosphate pathway, and lipopolysaccharide biosynthesis (Supplementary Figure S5B).

Among the differentially expressed genes identified, it is noteworthy that after the knockout of the *abaI* gene, three genes in the wzy polysaccharide synthesis pathway, *wza* (HKO16_RS18730), *wzb* (HKO16_RS18735), and *wzc* (HKO16_RS18740), were significantly down-regulated. Additionally, other genes related to capsular polysaccharide synthesis, such as *galU*, were also significantly down-regulated. However, the key gene *galM* involved in D-galactose metabolism was significantly up-regulated. These results suggest that after *abaI* gene knockout, the metabolism of galactose is enhanced, leading to a decrease in bacterial galactose content, which in turn results in reduced bacterial viscosity and attenuated toxicity. The top 20 genes with the most significant down-regulation in the transcriptome were screened (Supplementary Table S4). It was found that genes related to virulence such as glucosamine phosphorylase GlmM (HKO16_RS17815), a Zeta family protein (HKO16_RS17870), and the transcriptional regulator *BetI* (HKO16_RS04450), were significantly down-regulated following the knockout of the *abaI* gene, which may account for the reduced virulence observed. Furthermore, the *sul2* gene, related to antibiotic sensitivity, was also significantly down-regulated in the *abaI* knockout strain, indicating that the knockout of *abaI* may also affect bacterial resistance.

#### 3.3.2 RNA-Seq analysis of mouse lung infection

**Figure 8:**
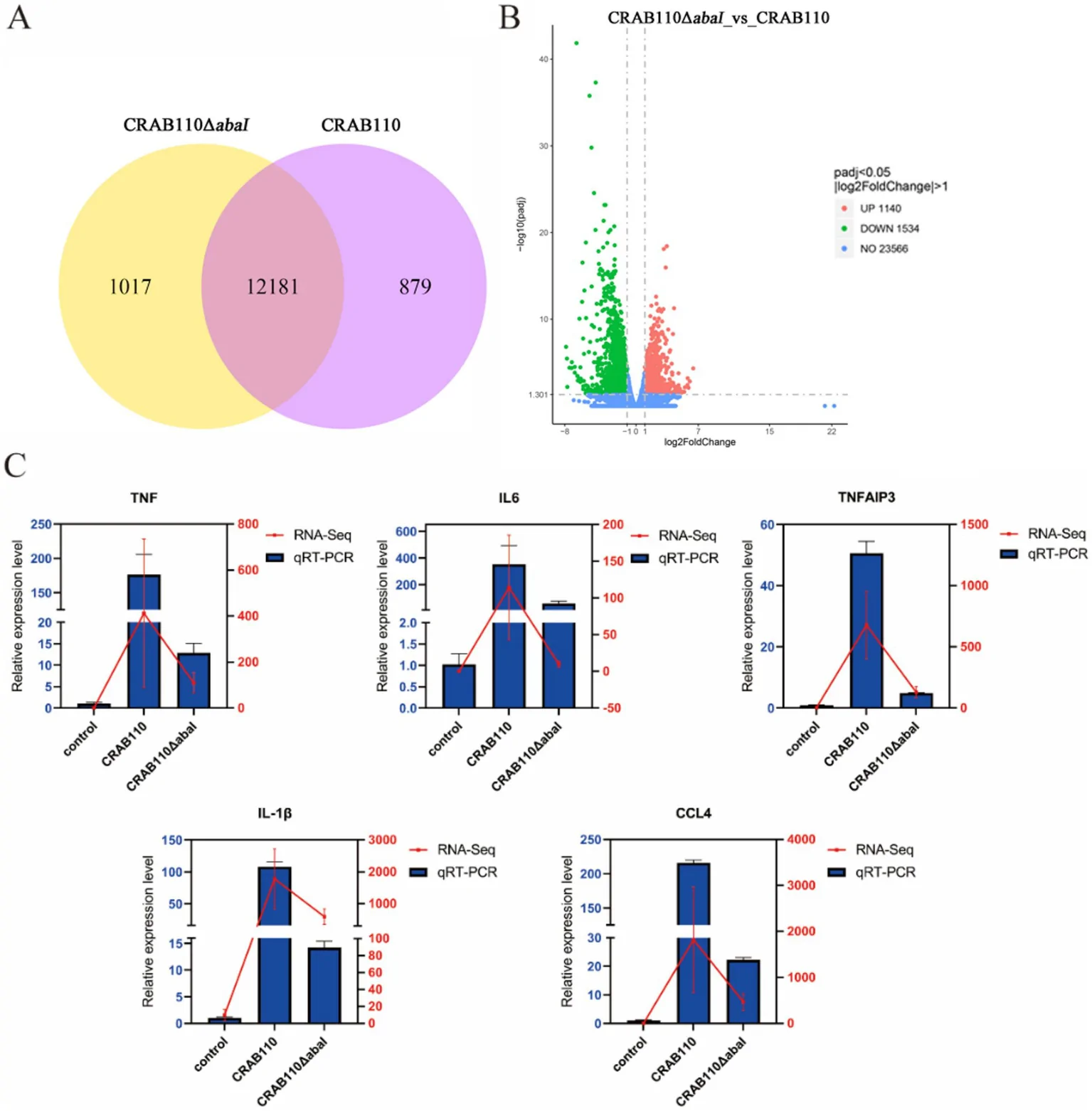
The impact of *abaI* gene knockout on the murine lung transcriptome. **(A)** Comparison of gene expression between the Knockout group, the Wild group, and the Saline group. **(B)** Volcano plot of differentially expressed genes between the Knockout and Wild groups. Red indicates gene upregulation, green indicates gene downregulation, and blue indicates no significant difference. **(C)** QRT-PCR validation. The relative expression levels of the five differentially expressed genes were determined, and the results are presented as the mean ± standard deviation from at least three biological replicates.

Mice were injected with CRAB110Δ*abaI*, CRAB110, and normal saline into their lungs, respectively. After 24 h, the lung tissues of the mice were collected for transcriptome sequencing. The results showed that 12,181 genes were co-expressed in the lungs of mice infected with CRAB110Δ*abaI* and CRAB110 (Figure 8A). Compared with CRAB110, CRAB110Δ*abaI* had 1,140 genes with significantly up-regulated expression and 1,534 genes with down-regulated expression (Figure 8B). In the wild-type group versus the saline group, 3,464 genes were up-regulated and 3,614 genes were down-regulated (Supplementary Figure S6A). In the knockout group versus the saline group, 1924 genes were up-regulated and 2011 genes were down-regulated (Supplementary Figure S6B). To validate the transcriptome results, five genes that were significantly expressed in all three transcriptomes and associated with inflammation (TNF, IL-6, TNFAIP3, IL-1β, CCL4) were chosen for qRT-PCR validation (Figure 8C). These results confirmed that the expression trends of these differential genes agreed with the high-throughput sequencing data, suggesting that the sequencing data are relatively accurate and reliable.

Differential genes between the CRAB110Δ*abaI* and the CRAB110 group were subjected to GO and KEGG analyses. The GO enrichment analysis revealed the top 30 pathways (Supplementary Figure S7A). These enrichments included various biological processes (BPs), such as response to bacteria, response to molecules of bacterial origin, response to lipopolysaccharides, positive regulation of cytokine production, leukocyte efflux, cytokine secretion, regulation of inflammatory response, cellular response to biological stimuli, and other processes associated with inflammation. Cellular Components (CCs) comprised membrane rafts, membrane microdomains, membrane regions, secretory granules, phagocytic vesicles, and extracellular organelles. Molecular Functions (MFs) involve receptor-ligand activity, cytokine activity, receptor regulatory activity, cytokine receptors, binding of cysteine-type endonuclease inhibitor peptide chains, and interleukin-1 receptor binding. The top thirty pathways obtained from KEGG enrichment, as shown in Supplementary Figure S7B, revealed that among the top 30 pathways related to infection and immunity enriched in the knockout versus wild-type group, the cytokine-cytokine receptor interaction pathway had the highest number of differentially expressed genes, most of which were down-regulated. In the pathways related to inflammation, including the IL-17, HIF-1, NF-κB, C-type lectin receptor, tumor necrosis factor, mitogen-activated protein kinase (MAPK), and Toll-like receptor signaling pathways, most genes showed a down-regulated trend in the CRAB110Δ*abaI* group. These results suggest that the IL-17 and NF-κB signaling pathways were differentially activated in mice infected with CRAB110ΔabaI compared with those infected with the wild-type strain CRAB110.

The GO and KEGG enrichments of the wild-type group vs. the saline control and the knockout group vs. the saline control are detailed in Supplementary Figures S8A–D. By combining the results of the GO and KEGG enrichments, we found that all three transcriptomes were enriched in two biological processes: response to bacteria and leukocyte migration. These processes correspond to a series of immune responses initiated by the host in response to bacterial invasion. The differentially expressed genes generated in response to host infection were primarily concentrated in the cellular membrane region. The molecular functions analysis revealed that these genes were associated mainly with receptor ligand activity, receptor regulatory activity, cytokine activity, cytokine receptor binding, and cytokine receptor activity. Moreover, all three groups were enriched in pathways such as cell-cytokine receptor interaction, the IL-17 signaling pathway, the NF-κB signaling pathway, and the tumor necrosis factor signaling pathway. This suggests that upon infection with *A. baumannii*, the host initiates these inflammatory pathways to combat the pathogen.

By screening genes involved in the three co-expressed transcriptomes within the significant pathways (Supplementary Table S5), which are related to immunity and infection, we identified genes such as Csf3, Slc39a14, and CXCL3 that were differentially expressed between the two infection groups and were annotated to be associated with innate immune response, immune cell differentiation, and migration. Additionally, genes such as *Ptgs2*, which are related to apoptosis (Li et al., 2022; Mao et al., 2017), interleukins responsible for the pro-inflammatory response (e.g., IL6, IL10, IL-1α, IL-1β, IL-17A, IL-17F), chemokines including CXCL1, CXCL2, CXCL3, CCL4, and CCL24, and tumor necrosis factor-related genes *TNF* and *TNFAIP3* were identified. Among these genes, compared with the saline group, the inflammation-related genes were significantly up-regulated in mice infected with CRAB110Δ*abaI*, although to a lesser extent than in the wild-type CRAB110 group. Conversely, compared with the CRAB110 infection group, these genes were significantly down-regulated in mice infected with CRAB110Δ*abaI*, indicating that *abaI* knockout attenuated the inflammatory response.

#### 3.3.3 Analysis of immune cell abundance in mice with lung infections

The distribution of 22 types of immune cells in different hosts under various infection conditions varies, suggesting that the immune microenvironments produced by the hosts also differ. The boxplot results (Supplementary Figure S9) indicate that the population of M1 macrophages in mice infected with the knockout strain CRAB110Δ*abaI* is greater than that in mice infected with the wild-type strain CRAB110, suggesting that the knockout strain CRAB110Δ*abaI* prompts the host to produce more M1 macrophages to combat the pathogen. Similarly, the population of neutrophils in mice infected with the knockout strain CRAB110Δ*abaI* is higher than that in mice infected with the wild-type strain CRAB110. Notably, the population of activated mast cells is greater in mice infected with the wild-type strain CRAB110 than in those infected with the knockout strain CRAB110Δ*abaI*. Among the 22 immune cells assessed, the activated mast cells are the most abundant, indicating that the wild-type strain CRAB110 recruits a significant number of mast cells to participate in the immune response upon infection. Collectively, these results suggest that the synergistic action of M1 macrophages, mast cells, and neutrophils aids the host in more effectively eliminating the disease caused by the knockout strain CRAB110Δ*abaI*.

## 4 Discussion

*A. baumannii* is an important opportunistic pathogen that can cause many infectious diseases. With the increasing number of multidrug-resistant *A. baumannii* (MRAB) and carbapenem-resistant *A. baumannii* (CRAB) isolates in clinical settings, infections caused by them pose a serious threat to the lives and health of patients (Aldali, 2023). Therefore, there is an urgent need to explore new therapeutic approaches to control *A. baumannii* infections. Bacterial virulence is a major determinant of health deterioration in infected patients, and the mucoid colony phenotype has been shown to correlate with virulence (Walker et al., 2019). Quorum Sensing (QS), an intercellular communication system used by bacteria to coordinate collective behavior, controls the virulence and pathogenicity of a wide range of microorganisms (Azimi et al., 2020). To investigate the function of the quorum-sensing gene *abaI* in regulating the virulence of *Acinetobacter baumannii*, the CRAB110 strain was utilized to construct an *abaI* knockout strain. Mucous production, virulence, and pathogenicity of the knockout strain were subsequently assessed, ultimately leading to the elucidation of the *abaI* gene’s role in virulence regulation.

The bacterial mucoid colony phenotype confers resistance to desiccation and phagocytosis, and the main component of the mucus is capsular polysaccharide (Luck et al., 2020). Studies have shown that CPS plays a vital role in strain virulence (He et al., 2023). In this study, after *abaI* was knocked out, the secretion of signaling molecules (acyl-homoserine lactones) was affected, suggesting that *abaI* is a key gene of QS (Figure 1D). In addition, this study demonstrated that the capsule production of the strain was significantly weakened following the *abaI* knockout. This finding suggests that knockout of the *abaI* gene affects mucilage production in *A. baumannii*. Congo red staining, CPS extraction, and density gradient centrifugation experiments all indicate that the capacity for capsule production is diminished after the *abaI* gene knockout. Moreover, since CPS is one of the crucial virulence factors in bacteria, the reduction in CPS content results in decreased virulence of the strain (Kaszowska et al., 2021). These results indicate that the virulence of CRAB110Δ*abaI* was significantly attenuated. This suggests that the *abaI* gene may enhance virulence by influencing capsule production, thereby increasing the pathogenicity of *A. baumannii*.

CPS formation in Gram-negative bacteria is primarily associated with the Wzy polysaccharide synthesis pathway (Kasimova et al., 2024). The wza-wzb-wzc complex plays a crucial role in synthesizing the Wzy polysaccharide. Among these, wza, an outer membrane protein encoded by the *wza* gene, is primarily responsible for transporting the capsular polysaccharide from the periplasmic space to the bacterial surface. *Wzb* acts as a phosphatase, anchoring the catalytic domain on the surface of *wzc*, dephosphorylating *wzc*, and controlling its phosphorylation and dephosphorylation, thus regulating the degree of polymerization and yield of the capsular polysaccharide (Niu et al., 2020). The transcriptome results of the knockout strain showed that the polysaccharide synthesis pathway genes (*wza*, *wzb*, and *wzc*) were significantly down-regulated. In addition, the *galU* gene, which is related to CPS synthesis (Ramón Roth et al., 2025), was also significantly down-regulated. Similar to the capsular polysaccharide composition observed in other bacteria, such as *Streptococcus thermophilus* MR-1C, which consists of repeating monomers of galactose, rhamnose, and fucose with galactose accounting for more than half of the composition (Robitaille et al., 2006). In this study, after knocking out the *abaI* gene, the capsule production of *A. baumannii* was reduced, and its virulence was also decreased. These findings suggest that the *abaI* gene knockout disrupts genes involved in capsular polysaccharides formation.

Inflammation is a host defense response to tissue damage; however, if the inflammatory stimulus persists, it may lead to tissue damage and organ dysfunction (Patrascu and Dumitru, 2025). The extensive and persistent infiltration of neutrophils and other inflammatory cells into the lungs is likely responsible for the tissue damage observed (Zeng et al., 2020). The pathological sections revealed that persistent infiltration of inflammatory cells in case of infection by CRAB110 results in damage to the organism, and the infection caused by CRAB110Δ*abaI* is more effectively cleared when the organism mounts an appropriate inflammatory response. Edema was also reduced in the CRAB110Δ*abaI* strain compared to the CRAB110 strain.

The chemokine family plays a central role in leukocyte recruitment to sites of inflammation. Typically, CXC chemokines are active on neutrophils and lymphocytes. In contrast, CC chemokines act on a variety of leukocyte subsets, including monocytes, eosinophils, dendritic cells, and natural killer cells, but not on neutrophils (Gray et al., 2023). It is reported that macrophages and mast cells can express CCL20, and the CCL20 is strongly chemotactic for lymphocytes (Zhai et al., 2023). CXCL5, also known as epithelial neutrophil-activating peptide 78 (ENA78), is an inflammatory mediator that has a strong chemotactic effect on neutrophils (Yildirim et al., 2018); CCL24, a pleiotropic chemokine, participates in inflammatory responses through the CCR3 receptor (Wang et al., 2023). In this study, the expression levels of *CCL20*, *CXCL5*, and *CCL24* were down-regulated in CRAB110Δ*abaI*-infected mice, suggesting that the inflammatory response induced by CRAB110Δ*abaI* in the host is weaker than that produced by CRAB110. These results indicate that the organism experienced an intense and sustained inflammatory response after infection with CRAB110, resulting in damage to the lung structure.

Mucosal epithelial cells, such as those in the respiratory tract and lung, serve as an essential defense line against pathogen infection. They can regulate the production of defense mediators, such as IL-8 and *β*-defensins, to recruit immune cells like neutrophils for the clearance of pathogens (Cerps et al., 2022). Macrophages are another type of innate immune cells that release chemokines to recruit other innate effector cells, including neutrophils locally, and independently kill pathogens. They also cooperate with complement to eliminate bacteria, playing a crucial role in the early defense against *A. baumannii* infection (Chen, 2020). Bacterial capsule polysaccharides (CPS) can evade phagocytosis by macrophages and are not easily eliminated by the complement, leading to further infection within the host (Talyansky et al., 2021). In this study, the *abaI* knockout strain exhibited a reduced mucoid phenotype and was more susceptible to macrophage clearance, indicating that knockout of the *abaI* gene attenuated the virulence of *A. baumannii*. According to immune cell infiltration analysis, more M1 macrophages and neutrophils were recruited in mice infected with CRAB110Δ*abaI* compared to those infected with wild-type CRAB110. Interestingly, it was found that many activated mast cells were produced in CRAB110-infected mice, whereas the mast cells produced in the CRAB110Δ*abaI*-infected mice were significantly reduced. These results indicate that mast cells also participate in the immune response.

IL-6 and TNF-*α* are important cytokines for the activation of the NF-κB inflammatory signaling pathway (Zhang et al., 2023; Schultheiss et al., 2022). In the present study, compared with the CRAB110 *-*infected mice, the serum level of IL-6 and TNF-α were lower in CRAB110Δ*abaI*-infected mice. Moreover, transcriptome and qRT-PCR analyses also showed that the IL-6 and TNF-α were down-regulated in CRAB110Δ*abaI*-infected mice. These results indicate that the *abaI* mutant can reduce the inflammatory response of *A. baumannii* in mice. IL-17A and IL-17F are known to induce the secretion of inflammatory cytokines such as IL-6, TNF-α, as well as CC and CXC subfamily chemokines. They can synergistically act with IL-1 and TNF to induce the expression of pro-inflammatory genes, which are associated with various inflammation-related diseases (Xu Y. et al., 2024; Jin et al., 2023). Among the numerous signaling pathways involved in bacteria-triggered inflammation in acute lung injury, the NF-κB signaling pathway is widely recognized as a central player (Zhu et al., 2023). In the present study, in comparison with A549 cells infected by CRAB110, the protein expression levels of IL-17 and p65 were down-regulated significantly in CRAB110Δ*abaI*. Analogously, compared with mice infected by CRAB110, it was found that the pro-inflammatory factors in IL-17 and NF-κB signaling pathways were also down-regulated in mice infected by CRAB110Δ*abaI*. In summary, these results suggest that IL-17 and NF-κB are the key pathways affected by *A. baumannii* infection.

## 5 Conclusion

**Figure 9:**
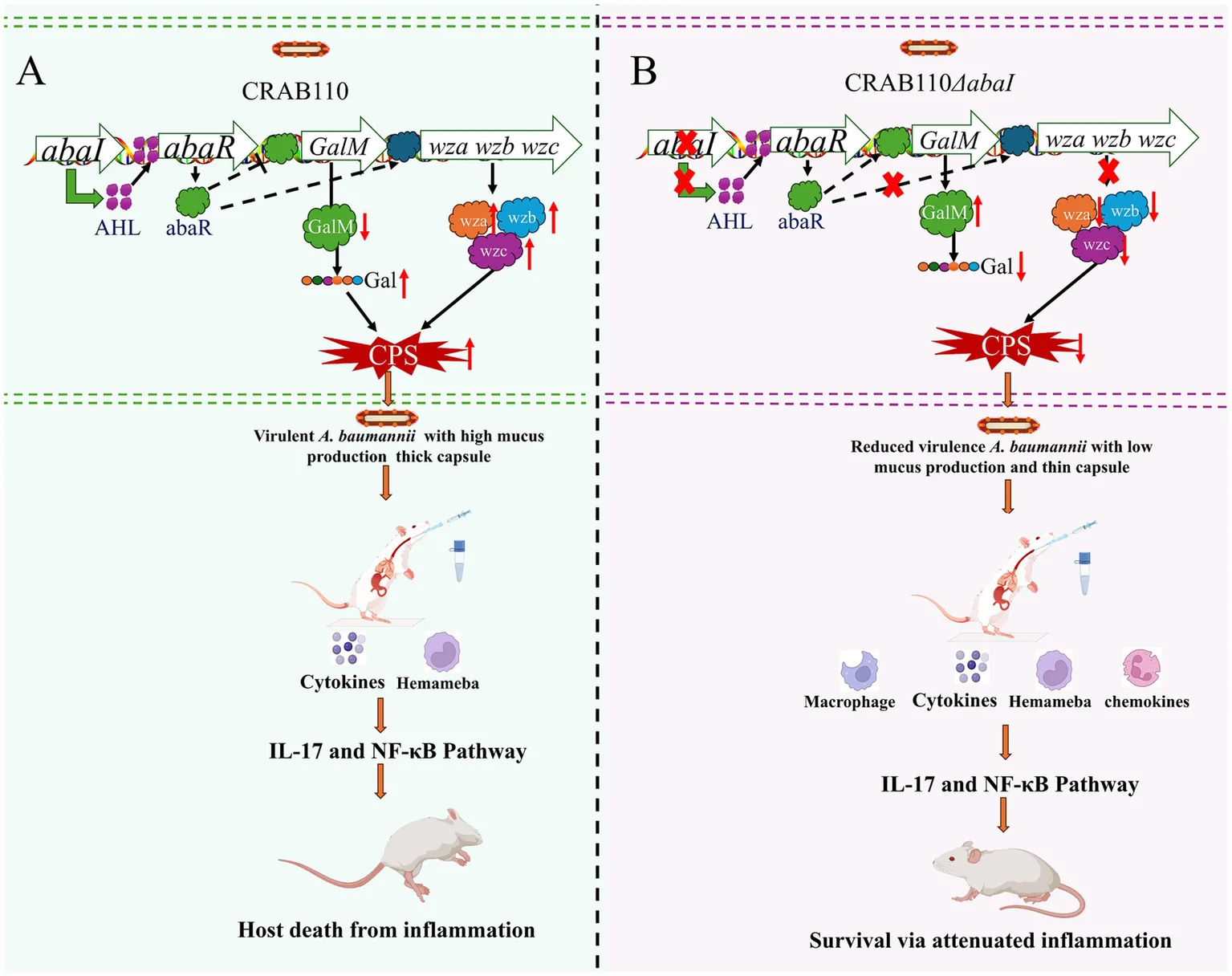
Mechanisms of *abaI* gene on capsule production in clinical strain CRAB110 and pathogenicity to the host. **(A)** In CRAB110, *abaI* gene expression drives acyl-homoserine lactone (AHL) production. AHL binds AbaR to indirectly upregulate capsule production-related genes and inhibit galactose catabolism, causing galactose accumulation in the capsule. Pulmonary infection of mice elevates their cytokines/chemokines, activates IL-17 and NF-κB pathways, and induces fatal excessive inflammation. **(B)** *abaI* knockout in CRAB110Δ*abaI* prevents AbaR-AHL binding, abrogating capsule assembly gene upregulation while activating galactose metabolism. This reduces bacterial mucoidy and virulence. Infection with CRAB110Δ*abaI* promotes cytokine production, increases macrophage recruitment and chemokine levels, attenuates the host inflammatory response, and enables mouse survival. A red upward arrow indicates that the gene is upregulated, and a downward arrow indicates that the gene is downregulated.

In the present study, we investigated the role of the *abaI* gene in capsule production and pathogenicity of *A. baumannii* strain CRAB110. The results demonstrated that *abaI* promotes the production of the signaling molecule acyl-homoserine lactone (AHL). In the wild-type strain CRAB110, genes associated with capsule formation (*wza*, *wzb*, and *wzc*) were upregulated, while galactose metabolism-related genes were downregulated, which may contribute to the formation of the mucoid phenotype. In a mouse pulmonary infection model, infection with CRAB110 elevated the levels of pro-inflammatory cytokines and chemokines and activated the IL-17 and NF-κB signaling pathways, leading to severe inflammation and death of the mice (Figure 9A). Knockout of the *abaI* gene (CRAB110Δ*abaI*) resulted in reduced mucoidy and attenuated virulence. Following infection with CRAB110Δ*abaI*, the host inflammatory response was attenuated, macrophage recruitment was enhanced, and the mice survived (Figure 9B). These findings suggest that the *abaI* gene plays an important role in regulating capsule production and virulence in *A. baumannii* and may represent a potential target for novel antimicrobial strategies.

## Data Availability

The original contributions presented in the study are publicly available. The RNA-seq data obtained in this study have been submitted to SRA with the accession number PRJNA1145176.
