# Supplementary material for: Quorum sensing gene abaI enhancing capsule production and pathogenicity of Acinetobacter baumannii
Source: Front Microbiol. 2026 Sep 1;17:1865788. doi: 10.3389/fmicb.2026.1865788 (PMC13574932; doi:10.3389/fmicb.2026.1865788)
Supplement: Supplementary file 1 [file Data_Sheet_1.DOCX]

**Figure S1 Construction of *abaI* knockout and complementation strains**

(A) Schematic diagram of CRISPR/Cas9 knockout. (B) PCR verification of the knockout strain. Lane 2 is the electrophoretic band of CRAB110 non-knockout bacteria, the size is around 400 bp, and Lane 3-6 is the electrophoretic band of CRAB110 knockout bacteria, the size is around 200 bp. (C) Amplification of the backfilled target gene. Lane 1 and 2 are target fragments. (D) Schematic diagram of the *abaI* gene ligated to pYMAb2. E: Validation of the complementation strain.

**Figure S2 The Impact of *abaI* Gene Knockout on Host Chemokines**

A-E are the assays of chemokines CXCL10, CXCL11, CCL12, CCL19, CCL27, and the control group is the lung tissue of mice injected with saline. **P <* 0.05, ***P* < 0.01, ****P* < 0.001, ns represents no statistical significance.

**Figure S3 Adhesion and phagocytosis experiments of the strains**

(A) A549 cell adhesion. (B) BEAS-2B cell adhesion. (C) RAW264.7 cell phagocytosis. ****P*< 0.001.

GAPDH-1	GAPDH-2

GAPDH-3	IL-17-1

IL-17-2	IL-17-3

p65-1	p65-2

P65-3

**Figure S4 IL-17 and p65 protein expression in lung epithelial cells A549.**

In these raw images, the samples in the three lanes of each picture from left to right are control, CRAB110, and CRAB110Δ*abaI*, respectively. The images selected for the main figure panels are GAPDH-1, IL-17-1, and p65-1.

**Figure S5 GO enrichment and KEGG enrichment of prokaryotic transcriptome differential genes**

(A) GO Enrichment Analysis, showing the top 20 most significantly enriched pathways. (B) KEGG Functional Enrichment Analysis, with an adjusted significance cutoff of *P* < 0.05, exhibits the top 6 most significantly enriched pathways.

**Figure S6 Volcano map of remaining differential genes in the mouse lung transcriptome**

(A) Wild group vs saline group differential gene volcano plot. (B) Differential gene volcano plot of knockout group vs saline group. Red color in the graph represents gene up-regulation, green color represents gene down-regulation, and blue color represents no differential.

**Figure S7 Enrichment bubble diagram of GO and KEGG in CRAB110Δ*abaI* vs CRAB110**

(A) Knockout group versus wild group GO enrichment plot: the results demonstrate the top 30 pathways with the most significant differences. (B) Knockout group versus wild group KEGG enrichment plot: the results demonstrate the top 30 pathways with the most significant differences.

**Figure S8 Comparative GO and KEGG Enrichment Profiles: CRAB110 vs. Control and CRAB110Δ*abaI* vs. Control**

(A) Bubble plots of GO enrichment in the wild group vs. the saline group. (B) Bubble plots of KEGG enrichment in the wild group vs. the saline group. (C) Bubble plots of GO enrichment in the knockout group vs. saline. (D) Bubble plots of KEGG enrichment in knockout group vs. saline.

**Figure S9 Abundance map of transcriptomic immune cells in mouse lungs**

The left ordinate represents the abundance ratio of immune cells, and the top *p* value represents whether the abundance of immune cells in the three groups is statistically different.
