# Supplementary material for: Quorum sensing gene abaI enhancing capsule production and pathogenicity of Acinetobacter baumannii
Source: Front Microbiol. 2026 Sep 1;17:1865788. doi: 10.3389/fmicb.2026.1865788 (PMC13574932; doi:10.3389/fmicb.2026.1865788)
Supplement: Supplementary file 2 [file Data_Sheet_2.DOCX]

**Table S1 Construction of the CRAB110 knockout strain Primer Information**

| Primer name | Primer sequence ((5’-3’) |
| --- | --- |
| *AbaI*-spacer-F | tagtATATTTCCCCAACTTCTCAA |
| *AbaI*-spacer-R | aaacTTGAGAAGTTGGGGAAATAT |
| *abaI*-ssDNA | TTGATGAAGTGCATCAACAATTACCGAAGATTCAGTTGCAATGATCTCGATTTATCACGTCTCATTTTGCTTGAAGAAGG |
| *abaI*-seq-F | TGTTGAATATTTGGGTTGGGA |
| *abaI*-seq-R | CATTGGTGGACCTGCACGATG |

**Table S2 Primers used for qPCR validation of prokaryotic transcriptome**

| Primer name | Primer sequence ((5’-3’) |
| --- | --- |
| HKO16_RS17575-F | TCTCTTTCCAGCAGAGCGTG |
| HKO16_RS17575-R | TGAAGCGGCCTTGACTATCG |
| HKO16_RS18735-F | TTTCGGGGTTGATTGGGCAT |
| HKO16_RS18735-R | GCTCGGCATTGAGCTTTCTC |
| HKO16_RS13995-F | AATCGCGCCTACTCTTCTCG |
| HKO16_RS13995-R | TCACTACTCGCCGCTGTTTT |
| HKO16_RS17835-F | TGTACGCTGAATGGTCGCTT |
| HKO16_RS17835-R | TTGGGGAACGAAGACGACAG |
| HKO16_RS17870-F | GTTCTACGTGGCGAAGGAGT |
| HKO16_RS17870-R | TAACGCCGGAGCATCTTCTT |
| 16S rRNA-F | TACACACCGCCCGTCACACC |
| 16S rRNA-R | CGGCTACCTTGTTACGACTTCACC |

**Table S3 Primers used for qPCR of mouse lung transcriptome**

| Primer name | Primer sequence ((5’-3’) |
| --- | --- |
| Tnf-F | ATGTCTCAGCCTCTTCTCATTC |
| Tnf-R | GCTTGTCACTCGAATTTTGAGA |
| IL6-F | CTCCCAACAGACCTGTCTATAC |
| IL6-R | CCATTGCACAACTCTTTTCTCA |
| Tnfaip3-F | CATAGAGACATGCCTCGAACTA |
| Tnfaip3-R | TAACCATTACACTTGGCATTGC |
| IL-1β-F | TCGCAGCAGCACATCAACAAG |
| IL-1β-R | TCCACGGGAAAGACACAGGTAG |
| CCL4-F | ACTTCCTGCTGTTTCTCTTACA |
| CCL4-R | CCAAGTCACTCATGTACTCAGT |
| GAPDH-F | AGGTCGGTGTGAACGGATTTG |
| GAPDH-R | TGTAGACCATGTAGTTGAGGTCA |

**Table S4 Top 20 most significantly down-regulated genes in the prokaryotic transcriptome**

| gene name | KO name | description | log2FC | Padj |
| --- | --- | --- | --- | --- |
| HKO16_RS17870 |  | zeta toxin family protein | -16.01 | 0.0000 |
| sul2 | sul2 | sulfonamide-resistant dihydropteroate synthase Sul2 | -13.95 | 0.0000 |
| HKO16_RS17835 |  | DDE-type integrase/transposase/recombinase | -11.66 | 0.0000 |
| HKO16_RS17865 |  | hypothetical protein | -11.19 | 50.0000 |
| HKO16_RS17875 |  | hypothetical protein | -10.67 | 0.0000 |
| HKO16_RS17860 | xerC | tyrosine-type recombinase/integrase | -9.34 | 0.0000 |
| HKO16_RS17880 |  | hypothetical protein | -8.81 | 0.0000 |
| HKO16_RS17850 |  | hypothetical protein | -7.68 | 0.0000 |
| HKO16_RS17815 | glmM | phosphoglucosamine mutase | -7.63 | 0.0000 |
| HKO16_RS17855 |  | hypothetical protein | -7.12 | 0.0000 |
| HKO16_RS01135 |  | TniQ family protein | -6.66 | 0.0000 |
| HKO16_RS17915 |  | TniQ famILy protein | -6.66 | 0.0000 |
| HKO16_RS17845 |  | hypothetical protein | -6.26 | 0.0000 |
| betI | betI | transcriptional regulator BetI | -5.83 | 0.0000 |
| HKO16_RS19255 |  | hypothetical protein | -5.54 | 0.0000 |
| betB | betB | betaine-aldehyde dehydrogenase | -5.49 | 0.0000 |
| HKO16_RS07260 |  | hypothetical protein | -5.45 | 0.0000 |
| betA | betA | choline dehydrogenase | -5.26 | 0.0000 |
| HKO16_RS18735 |  | low molecular weight phosphotyrosine protein phosphatase | -5.15 | 0.0000 |
| rpsI | rpsI | 30S ribosomal protein S9 | -4.74 | 0.0000 |

| **Table S5. Factors related to inflammatory response in mice infected with CRAB110Δ*abaI*, CRAB110, and the control group** | | | | | | | |
| --- | --- | --- | --- | --- | --- | --- | --- |
| **gene name** | **description** | **CRAB110Δ*abaI* vs control log2FC** | **Padj** | **CRAB110Δ*abaI* vs CRAB110 log2FC** | **Padj** | **CRAB110 vs control log2FC** | **Padj** |
| CXCL2 | chemokine (C-X-C motif) ligand 2 | 10.31527001 | 0 | -3.713739 | 0 | 14.02001469 | 0 |
| CXCL3 | chemokine (C-X-C motif) ligand 3 | 8.03559677 | 0 | -2.153592 | 0 | 10.18362525 | 0 |
| CXCL1 | chemokine (C-X-C motif) ligand 1 | 7.932413647 | 0 | -1.59247 | 0 | 9.514871628 | 0 |
| IL-6 | interleukin 6 | 8.044755862 | 0 | -3.496849 | 0 | 11.52535363 | 0 |
| CCL24 | chemokine (C-C motif) ligand 24 | 2.967222865 | 0 | -2.842293 | 0 | 5.79482508 | 0 |
| TNF | tumor necrosis factor | 7.343614686 | 0 | -1.879441 | 0.0008 | 9.211478159 | 0 |
| TNFAIP3 | tumor necrosis factor, alpha-induced protein 3 | 4.954113722 | 0 | -2.370322 | 0 | 7.311120998 | 0 |
| IL-1β | interleukin 1 beta | 5.9319318 | 0 | -1.559591 | 0.0011 | 7.479368916 | 0 |
| IL-1α | interleukin 1 alpha | 5.374018754 | 0 | -2.102487 | 0.0303 | 7.462214179 | 0 |
| CCL4 | chemokine (C-C motif) ligand 4 | 8.316694727 | 0 | -1.93319 | 0.0001 | 10.23624804 | 0 |
| IL-17A | interleukin 17A | 6.762989884 | 0.0001 | -3.610541 | 0 | 10.35793176 | 0 |
| IL-10 | interleukin 10 | 5.230603951 | 0 | -5.213209 | 0 | 10.42978748 | 0 |
| IL-17F | interleukin 17F | 3.768765689 | 0.0108 | -4.066806 | 0 | 7.824792445 | 0 |
| CCRL2 | chemokine (C-C motif) receptor-like 2 | 2.404783902 | 0 | -2.882086 | 0 | 5.273342469 | 0 |
| Mapk6 | mitogen-activated protein kinase 6 | 1.19603433 | 0 | -2.587027 | 0 | 3.769657962 | 0 |
| Csf3 | colony stimulating factor 3 (granulocyte) | 12.68238413 | 0 | -2.808952 | 0 | 15.47673829 | 0 |
| Slc39a14 | solute carrier family 39 (zinc transporter), member 14 | 3.853497011 | 0 | -1.138557 | 0.0001 | 4.980650718 | 0 |
| Ptgs2 | prostaglandin-endoperoxide synthase 2 | 2.430240936 | 0 | -3.628431 | 0.0001 | 6.044793533 | 0 |
